# A randomized trial of viral vector and adjuvanted protein HBV therapeutic vaccine in people with chronic hepatitis B on nucleos(t)ide analogs

**DOI:** 10.1097/HC9.0000000000001025

**Published:** 2026-08-20

**Authors:** Wen-Juei Jeng, Chi-Jen Chu, Apinya Leerapun, Anchalee Avihingsanon, Stefan Zeuzem, Stefan Bourgeois, Man-Fung Yuen, Manuel Romero-Gomez, Sheng-Shun Yang, Cristina Cravcenco, Bruno Salaun, Meng Shi, Ventzislav Vassilev, Joon Hyung Kim, Kristien Swinnen

**Affiliations:** 1 Department of Gastroenterology and Hepatology, Linkou Chang Gung Memorial Hospital, Taoyuan City, Taiwan; 2 College of Medicine, Chang Gung University, Taoyuan City, Taiwan; 3 Division of Gastroenterology and Hepatology, Department of Medicine, Taipei Veterans General Hospital, Taipei, Taiwan; 4 Faculty of Medicine, National Yang Ming Chiao Tung University, Taipei, Taiwan; 5 Department of Internal Medicine, Faculty of Medicine, Chiang Mai University, Chiang Mai, Thailand; 6 HIV-NAT, Thai Red Cross AIDS and Infectious Diseases Research Centre, and Center of Excellence in Tuberculosis, Faculty of Medicine, Chulalongkorn University, Bangkok, Thailand; 7 Department of Medicine, University Hospital, Goethe University Frankfurt, Frankfurt, Germany; 8 ZAS Cadix, Antwerp, Belgium; 9 Division of Gastroenterology and Hepatology, Department of Medicine, State Key Laboratory of Liver Research, The University of Hong Kong, Queen Mary Hospital, Hong Kong, China; 10 UCM Digestive Diseases, Virgen del Rocio University Hospital, Institute of Biomedicine of Seville (HUVR/CSIC/US), Seville, Spain; 11 Department of Medicine, University of Seville, Seville, Spain; 12 Division of Gastroenterology and Hepatology, Department of Internal Medicine, Kuang Tien General Hospital, Taichung, Taiwan; 13 GSK, Wavre, Belgium; 14 GSK, Rixensart, Belgium; 15 GSK, Rockville, Maryland, USA

**Keywords:** functional cure, “Hepatitis B Core Antigens”, “Hepatitis B Surface Antigens”, “Immunity, Cellular”, “Immunity, Humoral”, “Immunotherapy”

## Abstract

**Background:**

This study assessed the safety, efficacy, and immunogenicity of a therapeutic immunization strategy aimed at reaching a functional cure for chronic hepatitis B (CHB), relying on a heterologous prime-boost with viral vectors ChAd155-hIi-HBV and MVA-HBV, combined with sequential or concomitant administration of adjuvanted recombinant HBV proteins (HBc-HBs/AS01_B_).

**Methods:**

This single-blind, randomized, controlled, first-in-human, phase 1/2 trial enrolled adults aged 18–65 years with HBeAg-negative CHB, virally suppressed on nucleos(t)ide analogs (NAs), with HBsAg >50 IU/mL. Participants received NAs and the following regimens of 4 doses (8-week intervals): sequential administration of ChAd155-hIi-HBV, MVA-HBV, and 2 HBc-HBs/AS01_B_ doses; co-administration of ChAd155-hIi-HBV+HBc-HBs/AS01_B_, followed by 3 co-administered MVA-HBV+HBc-HBs/AS01_B_ doses; 4 HBc-HBs/AS01_B_ doses; 2 placebo doses followed by ChAd155-hIi-HBV and MVA-HBV administered alone or with HBc-HBs/AS01_B_; or 4 placebo doses. Safety, efficacy (≥1-log decrease in quantitative (q)HBsAg or HBsAg loss 24 weeks post-dose 4 [day (D)337]), antibody, and T-cell responses were evaluated.

**Results:**

In all, 134 participants were vaccinated. Grade 3 solicited adverse events (AEs) (median duration: 2–3 days) were more frequent after co-administration (systemic: 59.3%; administration-site: 33.3%) than sequential administration (systemic: 10.3%; administration-site: 12.8%) of high-dose viral vectors and proteins. No vaccine-related or fatal serious AEs were reported. After 4 doses, no participant had HBsAg loss or ≥1-log decrease in qHBsAg (D337 vs. D1). Co-administration induced the strongest anti-HBs response (73.7% achieved anti-HBs ≥10 mIU/mL 2 weeks post-dose 4 vs. 40.0% after sequential administration). Both sequential and co-administration induced HBc-specific CD4+ and CD8+ T-cell responses, with a prime-boost effect of the viral vectors.

**Conclusions:**

Heterologous prime-boost with ChAd155-hIi-HBV and MVA-HBV, combined with sequential or co-administration of HBc-HBs/AS01_B_, had an acceptable safety profile, were moderately immunogenic, but no participants showed the expected efficacy outcome.

## INTRODUCTION

HBV infection is a major global health challenge. Although HBV vaccination during infancy has strongly reduced the incidence of hepatitis B,^1^,^2^ the World Health Organization (WHO) estimated that 1.23 million new infections still occurred in 2022.^3^ According to WHO estimates, 254 million people were living with chronic hepatitis B in 2022, resulting in 1.1 million deaths, mainly due to severe liver diseases, including cirrhosis and hepatocellular carcinoma (HCC).^3^,^4^

Currently approved treatment options for chronic hepatitis B comprise pegylated interferon α and nucleos(t)ide analogs (NAs).^5^,^6^,^7^ While these treatments can effectively inhibit viral replication, they rarely reach a functional cure (defined as a sustained loss of HBsAg and undetectable HBV DNA in serum 24 weeks after treatment cessation).^7^,^8^,^9^ This is due to the persistence of covalently closed circular DNA in hepatocyte nuclei, which acts as a template for viral replication to sustain virion production, and chromosomally integrated HBV DNA, which continues to express HBsAg.^7^,^8^,^9^ The sustained presence of HBsAg contributes to the exhaustion and impairment of HBV-specific B-cell and CD4+/CD8+ T-cell immunity^9^,^10^,^11^ and is associated with an increased risk of HCC.^12^ Achieving a functional cure is currently considered the primary therapeutic goal and the preferred endpoint in clinical trials evaluating treatments for chronic hepatitis B.^7^,^8^,^9^ Novel treatment strategies under development aim to achieve a functional cure by reducing viral replication, lowering HBsAg levels, and/or restoring host anti-HBV immune responses.^13^,^14^ One such approach, therapeutic immunization, seeks to reverse immune exhaustion by inducing HBV-specific CD8+ T cells, the main effectors required for viral clearance, and HBV-specific CD4+ T cells, which also contribute to protective immunity.^10^,^11^,^15^,^16^

A therapeutic immunization strategy that relies on a heterologous prime-boost approach with 2 viral vector vaccines, combined with sequential or concomitant administration of AS01_B_-adjuvanted recombinant HBcAg and HBsAg proteins (HBc-HBs/AS01_B_), was developed. The viral vector used for priming was ChAd155-hIi-HBV, a chimpanzee-derived adenoviral vector encoding the human invariant chain (hIi, CD74), HBcAg, and HBsAg. Adenoviral vector vaccines encoding a target antigen fused with hIi have been shown to induce enhanced CD8+ T-cell responses in mice and non-human primates compared with vectors encoding the target antigen alone.^17^,^18^ The vector used for boosting is MVA-HBV, a modified vaccinia Ankara-based vector, also encoding HBcAg and HBsAg proteins. Adenoviral and MVA vectors have previously been shown to elicit strong CD8+ T-cell responses, especially when administered as a heterologous prime-boost immunization regimen,^19^,^20^,^21^ while adjuvanted proteins are known to induce robust antibody and CD4+ T-cell responses.^22^,^23^ The combination of these agents was therefore expected to restore a multifunctional HBV-specific immune response, which may reduce HBsAg levels and allow patients to discontinue NA therapy without virological or clinical relapse.

This first-in-human study aimed to assess the safety, efficacy, and immunogenicity of this immunization strategy in patients with chronic hepatitis B controlled on NA therapy. The study evaluated different dose levels of the vector and protein vaccines and assessed both sequential and concomitant administration of vectors and proteins. Findings are summarized in plain language in the Supplementary Material, https://links.lww.com/HC9/C451.

## METHODS

### Study design and participants

This single-blind, randomized, controlled, first-in-human, phase 1/2 trial (ClinicalTrials.gov: NCT03866187; EudraCT: 2017-001452-55/EUCT: 2023-510020-68-00) was performed in 8 countries (Belgium, France, Germany, Hong Kong, Spain, Taiwan, Thailand, and the United Kingdom). The study enrolled adults 18–65 years of age with HBeAg-negative chronic hepatitis B, adherent to and virally suppressed on treatment with an NA with a high barrier to resistance. Eligible individuals had an HBsAg concentration >50 IU/mL, were anti-HBs negative and anti-HBc positive, had normal ALT levels and HBV suppression within the 24 months before enrollment, with ALT ≤48 U/L and HBV DNA <10 IU/mL at screening, and had no cirrhosis or significant fibrosis (FibroScan transient elastography score <9.6 kPa and FibroTest score <0.59 at screening). Individuals who received immune-modifying drugs (including interferon), those with diabetes type I, those with evidence of HCV or HDV infection, and with suspicion of or confirmed HCC or any other liver cancer were excluded. Detailed inclusion and exclusion criteria are listed in the Supplemental Methods, https://links.lww.com/HC9/C451.

**FIGURE 1:**
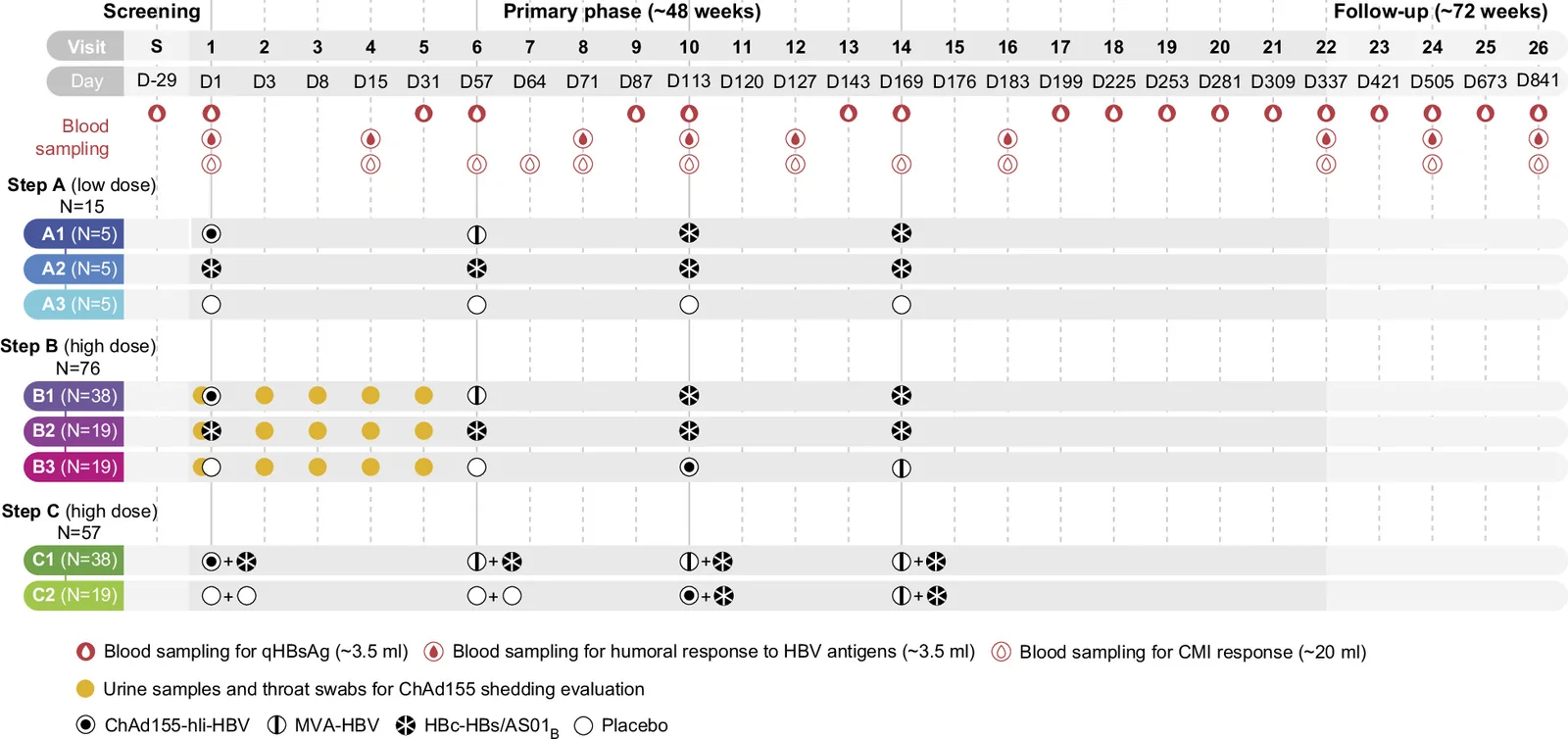
Study design. Enrollment in Steps A, B, and C occurred in a staggered manner, as detailed in the Supplemental Methods, https://links.lww.com/HC9/C451. Treatment with nucleos(t)ide analogs was maintained throughout the study. Efficacy proof-of-principle was evaluated on samples collected at D337. Follow-up was planned until D841 but was canceled after early study termination, which affected participants in Step C only. Visits 19 and 21 were canceled for Steps B and C. Blood sample collections at visits 5, 9, 13, and 17 were not mandatory. Immunogenicity analyses of D505 and D841 samples were canceled, except for the samples already processed for testing before study termination. Blood samples for CMI analyses were only collected in centers with access to peripheral blood mononuclear cell processing facilities. ChAd155-hIi-HBV, chimpanzee-derived adenoviral vector encoding the human invariant chain, HBcAg, and HBsAg; CMI, cell-mediated immunity; D, day; N, planned numbers of participants per group; these differ slightly from the actual numbers of enrolled or randomized participants shown in Figure 2; MVA-HBV, modified vaccinia Ankara-based vector encoding HBcAg and HBsAg; qHBsAg, quantitative HBsAg; S, screening.

The study had a staggered design with recruitment occurring in 3 steps (Supplemental Methods, https://links.lww.com/HC9/C451). Step A evaluated sequential administration of low doses of the viral vectors and proteins at 8-week intervals, with ChAd155-hIi-HBV (prime) given on day 1, MVA-HBV (boost) on day 57, and HBc-HBs/AS01_B_ on days 113 and 169 (group A1). Step A included 2 other groups: 1 receiving 4 low doses of HBc-HBs/AS01_B_ (group A2) and 1 receiving 4 placebo doses (group A3). Step B evaluated sequential administration of high doses of the viral vectors and proteins, with the same schedule as in Step A (group B1). Two other groups for Step B received either 4 high doses of HBc-HBs/AS01_B_ (on days 1, 57, 113, and 169, group B2) or 2 placebo doses (on days 1 and 57) followed by a prime-boost schedule of high doses of ChAd155-hIi-HBV (day 113) and MVA-HBV (day 169) (group B3). Step C evaluated co-administration of high doses of the viral vectors and proteins, with a priming dose of ChAd155-hIi-HBV co-administered with HBc-HBs/AS01_B_ on day 1, followed by 3 booster doses of MVA-HBV co-administered with HBc-HBs/AS01_B_ on days 57, 113, and 169 (group C1). Step C included a second group receiving a placebo on days 1 and 57, a ChAd155-hIi-HBV prime on day 113, and an MVA-HBV boost on day 169, both co-administered with HBc-HBs/AS01_B_ (group C2) (Figure 1). The investigational viral vector and protein vaccines are described in the Supplemental Methods, https://links.lww.com/HC9/C451.

Within each step, participants were randomized to the different groups, in a 1:1:1 ratio in Step A, a 2:1:1 ratio in Step B, and a 2:1 ratio in Step C. The randomization algorithm used a minimization procedure accounting for HBsAg concentration (<1000 IU/mL and ≥1000 IU/mL) measured at screening for participants in Steps B and C. Vaccines were administered in the deltoid muscle (of opposite arms in case of co-administration). Treatment with NAs was maintained throughout the study.

The primary phase of the study ended 24 weeks after the last immunization (day 337), at which point the immunization strategies’ efficacy proof-of-principle was evaluated. Participants were planned to be followed up for an additional 72 weeks, until day 841. However, the study was terminated early (during Step C) due to lack of efficacy; therefore, this additional follow-up was canceled for most participants enrolled in Step C. Planned study visits are presented in Figure 1.

An ancillary study (EudraCT: 2017-002574-39) was performed on participants from Step B to assess the potential shedding of ChAd155-hIi-HBV up to 1 month after intramuscular administration (day 31).

The safety of the participants was monitored by an internal safety review committee in collaboration with an external expert with clinical expertise in hepatology. The safety review committee determined whether any of the predefined study-holding rules were met or whether there were any other safety signals (Supplemental Methods, https://links.lww.com/HC9/C451). The protocols and protocol amendments of both studies were approved by the study centers’ independent ethics committees (Supplemental Methods, https://links.lww.com/HC9/C451). The studies were conducted in compliance with the guiding principles of the Declaration of Helsinki and International Council for Harmonization Good Clinical Practice guidelines. Written informed consent was obtained from each participant before any study procedures were performed.

### Objectives

The primary objective was to assess the safety of the different immunization regimens up to day 337. Secondary objectives were to evaluate the long-term safety up to day 841; decrease in quantitative HBsAg (qHBsAg) concentration and HBsAg loss at day 337; and immunogenicity in terms of anti-HBs antibodies, anti-HBc antibodies, and HBc-specific and HBs-specific CD4+ and CD8+ T cells (Supplemental Methods, https://links.lww.com/HC9/C451). The primary objective of the ancillary study was to assess ChAd155-hIi-HBV shedding.

### Safety assessments

The participants were instructed to record solicited administration-site and systemic adverse events (AEs) occurring within 7 days after each dose in diary cards. Unsolicited AEs starting within 30 days after each dose, and serious AEs (SAEs), medically attended AEs, potential immune-mediated diseases (pIMDs), liver disease-related and hematological AEs of special interest (AESIs), pregnancies, and AEs leading to withdrawal occurring from day 1 until study end were recorded in electronic case report forms.

Liver disease-related AESIs were defined as AEs related to the underlying chronic HBV infection and were characterized by ALT flares with or without other substantial biochemical changes, hepatic decompensation, and/or HBV DNA breakthrough (Supplemental Methods, https://links.lww.com/HC9/C451). Hematological AESIs were spontaneous local or systemic bleeding with thrombocytes <50,000 platelets/mm^3^, anemia with hemoglobin <9.5 g/dL, and thrombosis with thrombocytopenia syndrome (Supplemental Methods, https://links.lww.com/HC9/C451). pIMDs were AEs specified in a protocol-defined list and included autoimmune diseases and other inflammatory and/or neurologic disorders judged by the investigator as having an autoimmune origin.

The intensity of AEs was graded from 1 (mild) to 3 (severe), and the relationship between the intervention and the occurrence of each unsolicited AE was determined by the investigators using their clinical judgment. Hematological, biochemical, and urine analyses were performed to detect abnormalities within 30 days after each immunization, using toxicity grading from 1 (mild) to 4 (life-threatening).

### Efficacy assessment

To evaluate changes in HBsAg concentration in circulation, blood samples were to be collected from all participants before each dose, 1 month after each dose, every 4 weeks after the fourth dose until day 337, and every 12–24 weeks thereafter until study end (Figure 1). Analyses from day 505 onward were canceled after the study’s early termination. qHBsAg was determined with an electrochemiluminescence immunoassay (*Elecsys* HBsAg II quant II, Roche) on a cobas e immunoassay analyzer. The assay had a lower limit of quantification (LLOQ) of 0.05 IU/mL. HBsAg loss was defined as qHBsAg <LLOQ.

### Immunogenicity assessments

Blood samples to evaluate the humoral response to HBV antigens were planned to be collected from all participants before the first and third doses, 2 weeks after each dose, and 24, 48, and 96 weeks after the fourth dose (Figure 1). Blood samples for cell-mediated immunity assessments were to be collected before each dose, 2 weeks after each dose, 1 week after the second dose, and 24, 48, and 96 weeks after the fourth dose from all participants from sites with access to peripheral blood mononuclear cell (PBMC) processing facilities. After the early termination of the study, the immunogenicity analyses of day 505 and day 841 samples were canceled, except for the samples already processed for testing before termination.

Anti-HBs antibodies were measured using the ADVIA Centaur chemiluminescent immunoassay (Siemens Healthcare), with a limit of detection of 6.2 mIU/mL and LLOQ of 7.65 mIU/mL. Anti-HBc antibodies were measured on a subset of participants from B2 and C1 only (due to the study’s early termination) using a quantitative assay developed based on the Atellica IM HBcT2 commercial kit (Siemens), with an LLOQ of 0.53 IU/mL (Supplemental Methods, https://links.lww.com/HC9/C451).

To measure HBs-specific and HBc-specific CD4+ and CD8+ T-cell frequencies, PBMCs were stimulated in vitro with peptide pools covering the HBsAg or HBcAg sequences, immunostained for CD4 or CD8, CD3, and for various activation markers, the expression of which was then quantified using flow cytometry (Supplemental Methods, https://links.lww.com/HC9/C451). The LLOQ was 590 HBs-specific or HBc-specific CD4+ or CD8+ T cells per 10^6^ CD4+ or CD8+ T cells.

### ChAd155-hIi-HBV shedding assessment

Throat swabs and urine samples to assess ChAd155-hIi-HBV shedding were collected on days 1, 3, 8, 15, and 31 from the participants in the ancillary study. ChAd155 vector DNA was detected by quantitative PCR. Limits of detection were 371 copies/mL for throat swabs and 621 copies/mL for urine samples.

### Statistical analyses

We aimed to enroll 38 participants each in groups B1 and C1, and 19 each in groups B2, B3, and C2. For the ancillary study, we planned to enroll the 76 participants randomized in Step B of the main study (Supplemental Methods, https://links.lww.com/HC9/C451). All analyses were exploratory with no adjustments for multiplicity and were performed using SAS software version 9.4 or SAS Viya 4.0 (SAS Institute, Cary, NC, USA). Changes to the planned analyses because of early study termination are listed in the Supplemental Methods, https://links.lww.com/HC9/C451.

The efficacy proof-of-principle was informed by (1) at least 15% of participants (ie, lower limit of the 80% CI ≥15%) in one of the vaccine groups showing ≥1-log decrease in qHBsAg or HBsAg loss at day 337 versus day 1, or (2) at least a 1-log difference in mean HBsAg concentration between a vaccine group at day 337 and its control group at the relevant timepoint (day 113 or day 337 depending on the intervention), that is, lower limit of 80% CI on the adjusted ratio of HBsAg geometric mean concentrations (GMCs) in vaccine over control >1.

Safety was evaluated in the exposed set (all participants who received at least 1 dose). Percentages of participants reporting the different types of AEs were calculated with exact 95% CIs. Efficacy and immunogenicity were evaluated on the modified per-protocol sets (participants who received all 4 doses per protocol with a maximum interval of 111 days between doses, who complied with the allowed interval between dose 4 and day 337, who did not receive a product or present with a medical condition leading to exclusion from the modified per-protocol set, and who had post-immunization efficacy/immunogenicity results available for at least 1 timepoint/assay).

The qHBsAg-adjusted GMC ratio between groups was computed with 80% CIs using an ANCOVA model on the log_10_ transformation of the concentrations. The model included vaccine group and country as fixed effects and pre-immunization concentration as a covariate.

Anti-HBs and anti-HBc GMCs were calculated with exact 95% CIs by taking the anti-log of the mean of the log_10_ concentration transformations. The percentages of participants with anti-HBs antibody concentrations ≥10 mIU/mL and seropositivity rates for anti-HBc antibodies (defined as the percentage of participants with a concentration ≥0.53 IU/mL) were calculated with exact 95% CIs.

Frequencies (median, IQR, range) of HBc-specific and HBs-specific polypositive CD4+ and CD8+ T cells (defined as cells expressing at least 2 activation markers, of which at least one cytokine, among CD40 ligand, 4-1BB, interferon γ, tumor necrosis factor α, interleukin-2, interleukin-13, and interleukin-17) were calculated. The percentages of HBc-specific and HBs-specific polypositive CD4+ and CD8+ T-cell responders (defined as participants with a post-immunization/pre-immunization ratio in CD4+ or CD8+ polypositive T-cell frequencies above the maximum ratio observed in placebo controls) were calculated.

The percentages of participants with ChAd155 vector DNA equal to or greater than the limits of detection of the quantitative PCR assays in throat swabs and urine samples were calculated with exact 95% CIs. The B2 and B3 groups (used as control) were pooled for this analysis.

## RESULTS

### Study participants

**FIGURE 2:**
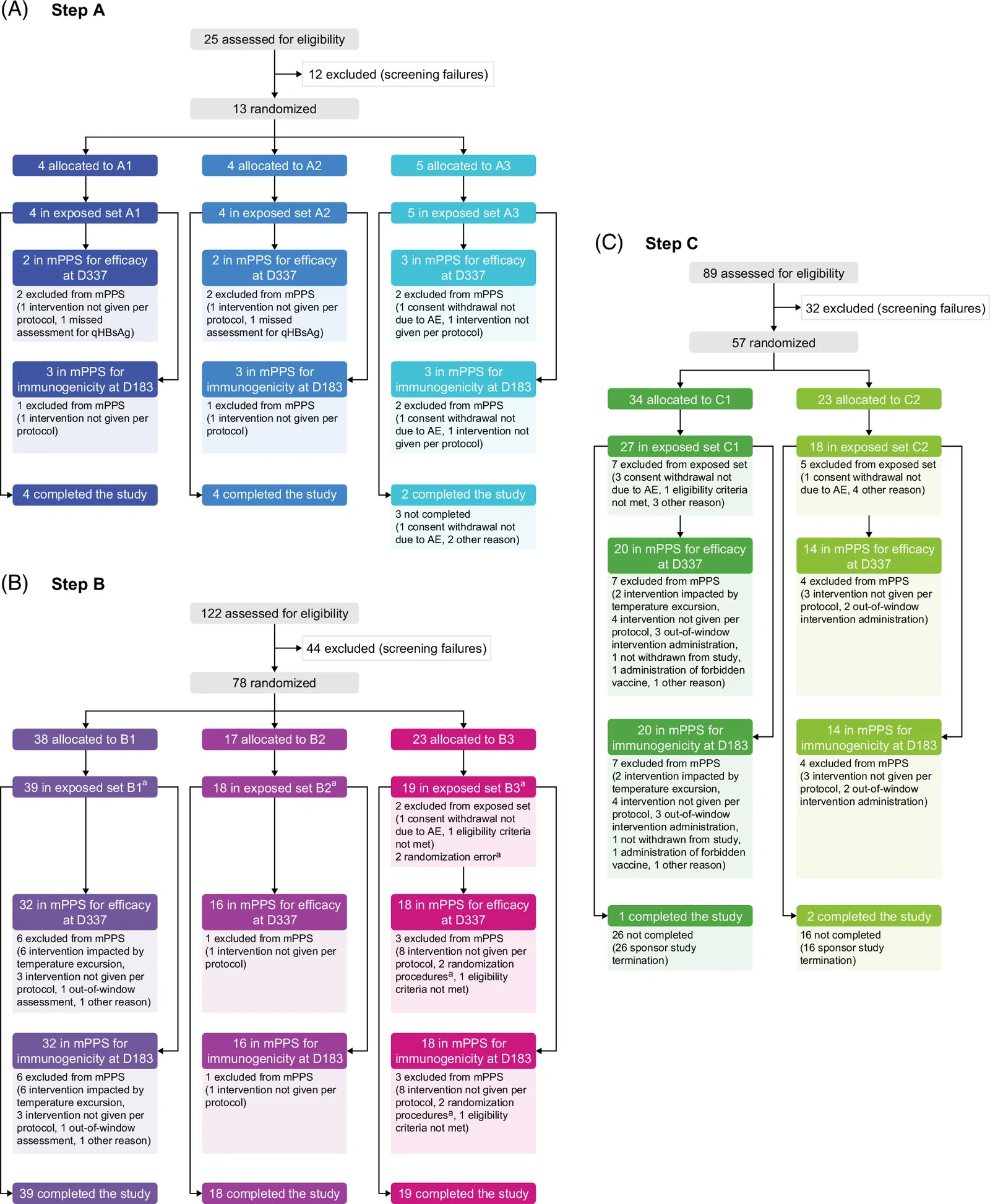
Flow of participants in Steps A, B, and C. For eliminations from the mPPS, multiple reasons could apply for 1 participant; all reasons are listed in the figure as the number of occurrences rather than the number of participants (eg, if a participant had multiple vaccine doses not administered per protocol, as well as a vaccine dose impacted by a temperature excursion, each of these is counted as an occurrence). AE, adverse event; D, day; mPPS, modified per-protocol set; N, number of participants in the enrolled, randomized, or exposed set, or number of participants who completed the study; n, number of participants in the mPPS; qHBsAg, quantitative HBsAg. ^a^Two participants were randomized to group B3 but received either the intervention for group B1 (n=1) or group B2 (n=1). These participants were included in the exposed set in the groups as per the administered intervention. Neither of these was part of the mPPS.

The study started on 28 March 2019 and was terminated early on 8 October 2024 due to lack of efficacy. The ancillary study was conducted between 27 October 2020 and 2 March 2022. Thirteen participants were randomized and included in the exposed set in Step A (4 each in A1 and A2, 5 in A3), 76 in Step B (39 in B1, 18 in B2, 19 in B3), and 45 in Step C (27 in C1, 18 in C2). Of these, 9 participants in Step A, 75 in Step B, and 41 in Step C received all 4 doses, 12 participants in Step A, 76 in Step B, and 45 in Step C attended the visit at day 337, and 10 participants in Step A, 76 in Step B, and 3 in Step C completed the study, with all withdrawals in Step C due to study termination (Figure 2).

In the exposed set, the mean age of the participants at baseline was 44.2 (SD 9.3) years in Step A, 49.9 (SD 8.1) years in Step B, and 45.9 (SD 8.4) years in Step C. Across the different steps, there were more male than female participants. In Step A, most participants were White; in Steps B and C, most were Asian. The majority of participants (84.6% in Step A, 65.8% in Step B, and 73.3% in Step C) had a qHBsAg ≥1000 IU/mL at screening (Table 1).

**TABLE 1:** Baseline characteristics of the participants (exposed set)

	Step A (sequential administration of low doses)				Step B (sequential administration of high doses)				Step C (co-administration of high doses)		
Characteristic	A1, N=4	A2, N=4	A3, N=5	Total, N=13	B1, N=39	B2, N=18	B3, N=19	Total, N=76	C1, N=27	C2, N=18	Total, N=45
Mean±SD age on day 1, years	42.8±3.2	48.5±9.7	41.8±12.4	44.2±9.3	48.8±8.9	51.3±6.8	50.8±7.7	49.9±8.1	44.4±7.9	48.3±8.8	45.9±8.4
Female sex, n (%)	2 (50.0)	1 (25.0)	1 (20.0)	4 (30.8)	9 (23.1)	3 (16.7)	5 (26.3)	17 (22.4)	12 (44.4)	2 (11.1)	14 (31.1)
Race, n (%)											
Asian	1 (25.0)	0 (0.0)	1 (20.0)	2 (15.4)	20 (51.3)	9 (50.0)	11 (57.9)	40 (52.6)	18 (66.7)	11 (61.1)	29 (64.4)
Black/African American	0 (0.0)	2 (50.0)	1 (20.0)	3 (23.1)	6 (15.4)	1 (5.6)	0 (0.0)	7 (9.2)	2 (7.4)	2 (11.1)	4 (8.9)
White	3 (75.0)	2 (50.0)	3 (60.0)	8 (61.5)	12 (30.8)	8 (44.4)	8 (42.1)	28 (36.8)	7 (25.9)	5 (27.8)	12 (26.7)
Other	0 (0.0)	0 (0.0)	0 (0.0)	0 (0.0)	1 (2.6)	0 (0.0)	0 (0.0)	1 (1.3)	0 (0.0)	0 (0.0)	0 (0.0)
qHBsAg at screening, n (%)											
<1000 IU/mL	0 (0.0)	0 (0.0)	2 (40.0)	2 (15.4)	13 (33.3)	6 (33.3)	7 (36.8)	26 (34.2)	7 (25.9)	5 (27.8)	12 (26.7)
≥1000 IU/mL	4 (100)	4 (100)	3 (60.0)	11 (84.6)	26 (66.7)	12 (66.7)	12 (63.2)	50 (65.8)	20 (74.1)	13 (72.2)	33 (73.3)
qHBsAg on day 1											
Geometric mean (95% CI), IU/mL	1461 (330–6475)	1942 (380–9926)	758 (189–3042)	NC	1292 (731–2284)	901 (479–1695)	846 (419–1709)	NC	1637 (843–3181)	1375 (633–2988)	NC
50–100 IU/mL, n (%)	0 (0.0)	0 (0.0)	0 (0.0)	0 (0.0)	3 (7.7)	0 (0.0)	1 (5.3)	4 (5.3)	2 (7.4)	1 (5.6)	3 (6.7)
100–200 IU/mL, n (%)	0 (0.0)	0 (0.0)	1 (20.0)	1 (7.7)	4 (10.3)	3 (16.7)	2 (10.5)	9 (11.8)	0 (0.0)	2 (11.1)	2 (4.4)
200–1000 IU/mL, n (%)	1 (25.0)	2 (50.0)	2 (40.0)	5 (38.5)	11 (28.2)	5 (27.8)	7 (36.8)	23 (30.3)	9 (33.3)	4 (22.2)	13 (28.9)
>1000 IU/mL, n (%)	3 (75.0)	2 (50.0)	2 (40.0)	7 (53.8)	21 (53.8)	10 (55.6)	9 (47.4)	40 (52.6)	16 (59.3)	11 (61.1)	27 (60.0)
Mean±SD disease duration, years	24.0±11.3	17.0±17.0	16.8±10.7	19.1±12.4	19.8±9.9	23.6±10.8	24.1±9.6	21.8±10.1	18.3±8.8	23.7±12.7	20.4±10.7
Choice of NA, n (%)											
ETV	2 (50.0)	3 (75.0)	2 (40.0)	7 (53.8)	19 (48.7)	7 (38.9)	11 (57.9)	37 (48.7)	14 (51.9)	7 (38.9)	21 (46.7)
Emtricitabine, TDF	0 (0.0)	0 (0.0)	0 (0.0)	0 (0.0)	0 (0.0)	1 (5.6)	0 (0.0)	1 (1.3)	0 (0.0)	0 (0.0)	0 (0.0)
TAF	0 (0.0)	0 (0.0)	0 (0.0)	0 (0.0)	4 (10.3)	0 (0.0)	1 (5.3)	5 (6.6)	5 (18.5)	2 (11.1)	7 (15.6)
TDF	1 (25.0)	1 (25.0)	2 (40.0)	4 (30.8)	8 (20.5)	5 (27.8)	6 (31.6)	19 (25.0)	4 (14.8)	7 (38.9)	11 (24.4)
Not specifiable	0 (0.0)	0 (0.0)	0 (0.0)	0 (0.0)	1 (2.6)	0 (0.0)	0 (0.0)	1 (1.3)	0 (0.0)	0 (0.0)	0 (0.0)
Mean±SD FibroScan, kPa	5.4±2.0	5.2±1.2	4.9±1.5	5.2±1.5	5.0±1.2	4.9±1.5	5.5±1.7	5.1±1.4	5.1±1.3	5.6±1.6	5.3±1.4
Mean±SD FibroTest score	0.4±0.1	0.2±0.1	0.3±0.1	0.3±0.1	0.3±0.1	0.4±0.1	0.3±0.1	0.4±0.1	0.2±0.1	0.4±0.2	0.3±0.1

Interventions administered: A1, low doses of ChAd155-hIi-HBV (prime) on day 1, MVA-HBV (boost) on day 57, and HBc-HBs/AS01_B_ on days 113 and 169; A2, 4 low doses of HBc-HBs/AS01_B_ (days 1, 57, 113, and 169); A3, 4 placebo doses (days 1, 57, 113, and 169); B1, high doses of ChAd155-hIi-HBV (prime) on day 1, MVA-HBV (boost) on day 57, and HBc-HBs/AS01_B_ on days 113 and 169; B2, 4 high doses of HBc-HBs/AS01_B_ (days 1, 57, 113, and 169); B3, 2 placebo doses (days 1 and 57), followed by high doses of ChAd155-hIi-HBV (prime) on day 113 and MVA-HBV (boost) on day 169; C1, high dose of ChAd155-hIi-HBV (prime) on day 1 followed by 3 high doses of MVA-HBV (boost) on days 57, 113, and 169, with all 4 doses co-administered with high doses of HBc-HBs/AS01_B_; C2, 2 placebo doses (days 1 and 57), followed by high doses of ChAd155-hIi-HBV (prime) on day 113 and MVA-HBV (boost) on day 169, both co-administered with high doses of HBc-HBs/AS01_B_. ETV, entecavir; n (%), number (percentage) of participants with the indicated characteristic; N, number of participants in the exposed set; NA, nucleos(t)ide analog; NC, not calculated; qHBsAg, quantitative HBsAg; SD, standard deviation; TAF, tenofovir alafenamide; TDF, tenofovir disoproxil fumarate.

Of the 76 participants randomized in Step B, 53 were enrolled in the ancillary study, with 52 included in the exposed set (24 in B1 who received ChAd155-hIi-HBV and 28 in the pooled control group). The mean age of these participants was 49.2 (SD 7.8) years, 76.9% were male, 48.1% were White, and 46.2% were Asian.

### Safety

**FIGURE 3:**
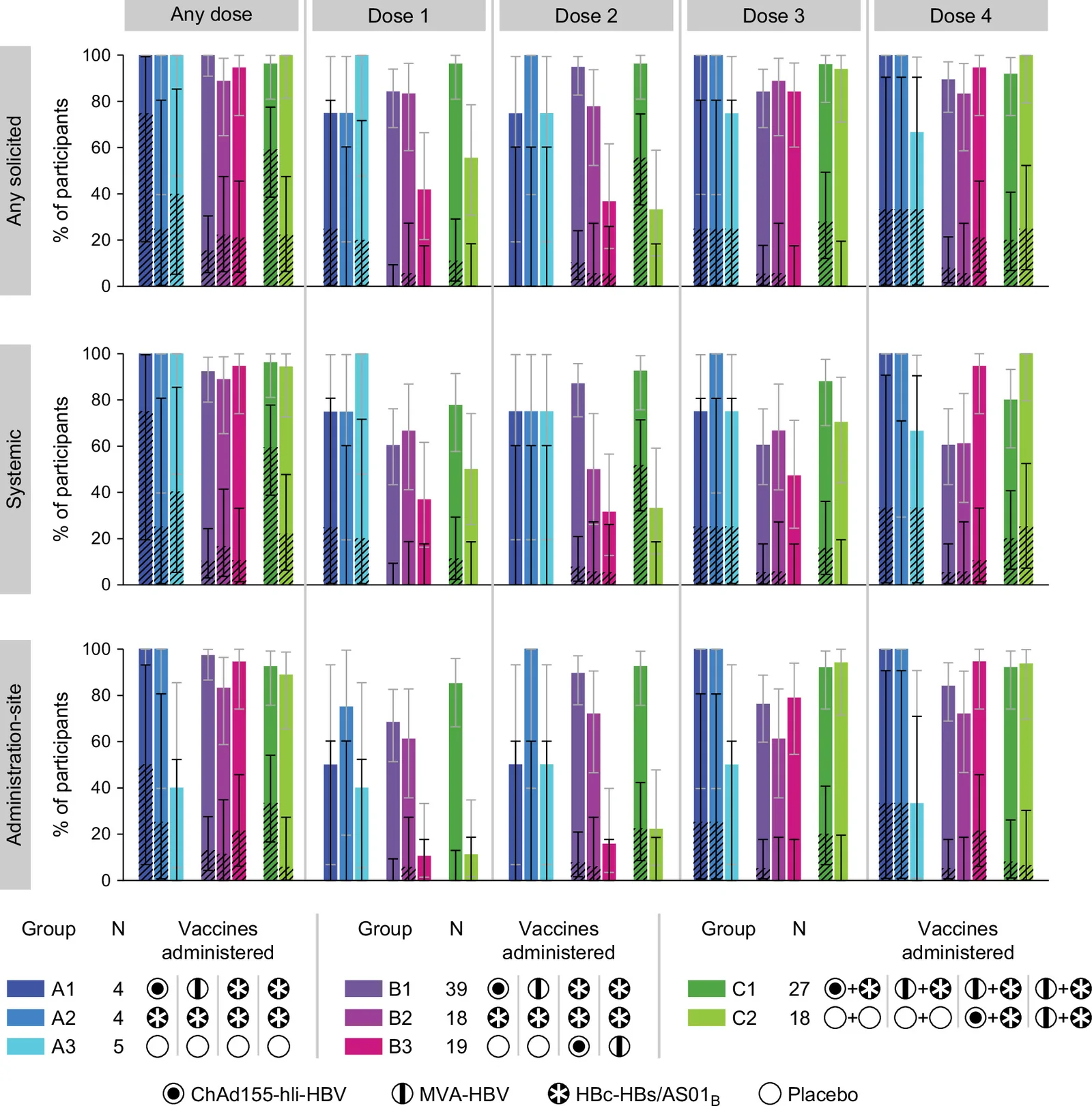
Percentage of participants reporting solicited adverse events with onset within 7 days post-immunization overall and after each dose (exposed set). Hashed bars indicate the proportion of participants reporting grade 3 solicited adverse events. Error bars depict 95% confidence intervals. Interventions administered: A1, low doses of ChAd155-hIi-HBV (prime) on day 1, MVA-HBV (boost) on day 57, and HBc-HBs/AS01_B_ on days 113 and 169; A2, 4 low doses of HBc-HBs/AS01_B_ (days 1, 57, 113, and 169); A3, 4 placebo doses (days 1, 57, 113, and 169); B1, high doses of ChAd155-hIi-HBV (prime) on day 1, MVA-HBV (boost) on day 57, and HBc-HBs/AS01_B_ on days 113 and 169; B2, 4 high doses of HBc-HBs/AS01_B_ (days 1, 57, 113, and 169); B3, 2 placebo doses (days 1 and 57), followed by high doses of ChAd155-hIi-HBV (prime) on day 113 and MVA-HBV (boost) on day 169; C1, high dose of ChAd155-hIi-HBV (prime) on day 1 followed by 3 high doses of MVA-HBV (boost) on days 57, 113, and 169, with all 4 doses co-administered with high doses of HBc-HBs/AS01_B_; C2, 2 placebo doses (days 1 and 57), followed by high doses of ChAd155-hIi-HBV (prime) on day 113 and MVA-HBV (boost) on day 169, both co-administered with high doses of HBc-HBs/AS01_B_. N, number of participants with available data; differed post-dose 1 for B1 (N=38), post-dose 2 for A3 (N=4), post-dose 3 for A3 (N=4), B1 (N=38), C1 (N=25), C2 (N=17), post-dose 4 for A1 (N=3), A2 (N=3), A3 (N=3), B1 (N=38), C1 (N=25), C2 (N=16).

Most participants across groups experienced at least one solicited systemic or administration-site AE within 7 days after at least one of the doses (Figure 3). The median duration of solicited AEs was 2–7 days across most groups. In Step A, 75.0% (3 out of 4, A1), 25.0% (1/4, A2), and 40.0% (2/5, A3) of participants reported grade 3 solicited systemic AEs, and 50.0% (2/4, A1), 25.0% (1/4, A2), and 0.0% (0/5, A3) reported grade 3 administration-site AEs. In Step B, 10.3% (4/39, B1), 16.7% (3/18, B2), and 10.5% (2/19, B3) of participants reported grade 3 systemic AEs, and 12.8% (5/39, B1), 11.1% (2/18, B2), and 21.1% (4/19, B3) reported grade 3 administration-site AEs. In Step C, reporting rates for grade 3 solicited AEs were 59.3% (16/27, C1) and 22.2% (4/18, C2) for systemic, and 33.3% (9/27, C1) and 5.6% (1/18, C2) for administration-site AEs (Figure 3). The median duration of grade 3 solicited AEs was 2–3 days across groups, and none lasted longer than 4 days. The proportion of participants reporting solicited AEs did not increase with subsequent doses (Figure 3).

Unsolicited AEs starting within 30 days post-immunization were observed in 42.1%–100% of participants across groups (Table 2). Unsolicited AEs judged by the investigator as related to immunization were reported in 17.9% (7/39) of B1 participants, 5.6% (1/18) of B2 participants, 14.8% (4/27) of C1 participants, and 5.6% (1/18) of C2 participants (Table 2, footnote).

**TABLE 2:** Number and percentage of participants reporting adverse events (exposed set)

	Step A (sequential administration of low doses)			Step B (sequential administration of high doses)			Step C (co-administration of high doses)	
Type of AE	A1, N=4	A2, N=4	A3, N=5	B1, N=39	B2, N=18	B3, N=19	C1, N=27	C2, N=18
AEs until 30 days post-immunization, n (%, 95% CI)								
Any unsolicited	2 (**50.0**, 6.8–93.2)	4 (**100**, 39.8–100)	4 (**80.0**, 28.4–99.5)	24 (**61.5**, 44.6–76.6)	10 (**55.6**, 30.8–78.5)	8 (**42.1**, 20.3–66.5)	15 (**55.6**, 35.3–74.5)	9 (**50.0**, 26.0–74.0)
Grade 3 unsolicited^a^	0 (**0.0**, 0.0–60.2)	0 (**0.0**, 0.0–60.2)	0 (**0.0**, 0.0–52.2)	1 (**2.6**, 0.1–13.5)	0 (**0.0**, 0.0–18.5)	0 (**0.0**, 0.0–17.6)	0 (**0.0**, 0.0–12.8)	0 (**0.0**, 0.0–18.5)
Related unsolicited^b^	0 (**0.0**, 0.0–60.2)	0 (**0.0**, 0.0–60.2)	0 (**0.0**, 0.0–52.2)	7 (**17.9**, 7.5–33.5)	1 (**5.6**, 0.1–27.3)	0 (**0.0**, 0.0–17.6)	4 (**14.8**, 4.2–33.7)	1 (**5.6**, 0.1–27.3)
AEs until study end, n (%, 95% CI)								
Medically attended	2 (**50.0**, 6.8–93.2)	2 (**50.0**, 6.8–93.2)	1 (**20.0**, 0.5–71.6)	19 (**48.7**, 32.4–65.2)	9 (**50.0**, 26.0–74.0)	9 (**47.4**, 24.4–71.1)	12 (**44.4**, 25.5–64.7)	7 (**38.9**, 17.3–64.3)
Any SAE	0 (**0.0**, 0.0–60.2)	0 (**0.0**, 0.0–60.2)	0 (**0.0**, 0.0–52.2)	2 (**5.1**, 0.6–17.3)	0 (**0.0**, 0.0–18.5)	0 (**0.0**, 0.0–17.6)	3 (**11.1**, 2.4–29.2)	1 (**5.6**, 0.1–27.3)
Any pIMD	0 (**0.0**, 0.0–60.2)	0 (**0.0**, 0.0–60.2)	0 (**0.0**, 0.0–52.2)	0 (**0.0**, 0.0–9.0)	0 (**0.0**, 0.0–18.5)	0 (**0.0**, 0.0–17.6)	1 (**3.7**, 0.1–19.0)	0 (**0.0**, 0.0–18.5)
Any AESI	0 (**0.0**, 0.0–60.2)	0 (**0.0**, 0.0–60.2)	1 (**20.0**, 0.5–71.6)	1 (**2.6**, 0.1–13.5)	0 (**0.0**, 0.0–18.5)	0 (**0.0**, 0.0–17.6)	4 (**14.8**, 4.2–33.7)	0 (**0.0**, 0.0–18.5)
Liver disease AESI	0 (**0.0**, 0.0–60.2)	0 (**0.0**, 0.0–60.2)	1 (**20.0**, 0.5–71.6)	1 (**2.6**, 0.1–13.5)	0 (**0.0**, 0.0–18.5)	0 (**0.0**, 0.0–17.6)	2 (**7.4**, 0.9–24.3)	0 (**0.0**, 0.0–18.5)
Hematological AESI	0 (**0.0**, 0.0–60.2)	0 (**0.0**, 0.0–60.2)	0 (**0.0**, 0.0–52.2)	0 (**0.0**, 0.0–9.0)	0 (**0.0**, 0.0–18.5)	0 (**0.0**, 0.0–17.6)	1 (**3.7**, 0.1–19.0)	0 (**0.0**, 0.0–18.5)

Interventions administered: A1, low doses of ChAd155-hIi-HBV (prime) on day 1, MVA-HBV (boost) on day 57, and HBc-HBs/AS01_B_ on days 113 and 169; A2, 4 low doses of HBc-HBs/AS01_B_ (days 1, 57, 113, and 169); A3, 4 placebo doses (days 1, 57, 113, and 169); B1, high doses of ChAd155-hIi-HBV (prime) on day 1, MVA-HBV (boost) on day 57, and HBc-HBs/AS01_B_ on days 113 and 169; B2, 4 high doses of HBc-HBs/AS01_B_ (days 1, 57, 113, and 169); B3, 2 placebo doses (days 1 and 57), followed by high doses of ChAd155-hIi-HBV (prime) on day 113 and MVA-HBV (boost) on day 169; C1, high dose of ChAd155-hIi-HBV (prime) on day 1 followed by 3 high doses of MVA-HBV (boost) on days 57, 113, and 169, with all 4 doses co-administered with high doses of HBc-HBs/AS01_B_; C2, 2 placebo doses (days 1 and 57), followed by high doses of ChAd155-hIi-HBV (prime) on day 113 and MVA-HBV (boost) on day 169, both co-administered with high doses of HBc-HBs/AS01_B_. AE, adverse event; AESI, adverse event of special interest; n (%), number (percentage) reporting the AE at least once; N, number of participants in the exposed set; pIMD, potential immune-mediated disease; SAE, serious adverse event.

^a^ The grade 3 unsolicited AE was not considered related to immunization by the investigator.

^b^ Unsolicited AEs considered related to immunization by the investigator were: influenza-like illness in 2 participants, asthenia in 2 participants, mouth ulceration (3 events in 1 participant), nausea, fatigue, and feeling hot in 1 participant each in the B1 group; influenza-like illness and tachycardia in 1 B2 participant; hematuria, proteinuria, asthenia, pyrexia, decreased neutrophil count, and rash in 1 participant each in C1; and hematuria and proteinuria in 1 participant in C2.

No SAEs were reported in Step A (Table 2 and Supplemental Table S1, https://links.lww.com/HC9/C451). Throughout the study, 3 SAEs were reported in the B1 group (gastric ulcer and esophageal ulcers in 1 participant and HCC with an Ishak modified fibrosis score of 3 at 2 weeks after diagnosis in another one), 3 in the C1 group (elevated HBV DNA, upper respiratory tract infection, and mycoplasma pneumonia in 1 participant each), and 1 in the C2 group (enlarged prostate). None of these were considered by the investigators to be related to the administered vaccines, and none were fatal. One participant in the C1 group experienced a pIMD of myocarditis that was judged to be related to immunization by the investigator. The event started 70 days after dose 1 (co-administration of ChAd155-hIi-HBV and HBc-HBs/AS01_B_) and resolved within 36 days. Liver disease-related AESIs were reported in 1 participant in A3 (hepatitis B), 1 in B1 (hepatitis B), and 2 in C1 (hepatitis B and elevated HBV DNA). All resolved and were of mild or moderate intensity. The hepatitis B case in B1, which started 245 days post-dose 4, was judged as related to immunization by the investigator. One participant in C1 experienced a hematological AESI (anemia), which was of mild intensity, resolved, and was considered unrelated to immunization. No participant withdrew due to an AE, but immunization was discontinued in the participant with the vaccine-related myocarditis event.

Most participants across groups had hematological and biochemical parameters within normal ranges at all assessed timepoints, and any reported abnormalities were predominantly of grade 1 or 2 intensity. One grade 4 abnormality (neutrophil decrease) was detected in a participant in B2.

Two safety signals were investigated, during which the study was put on a temporary hold: one to assess cardiac events (based on the aforementioned pIMD of myocarditis) and another one to evaluate the occurrence of proteinuria and hematuria (based on biochemical parameters collected during the study). An in-depth review of clinical trial data and published literature concluded that none of these events represented a new safety risk for the participants and that the anticipated benefit–risk profile of the study treatments remained favorable. Hence, the study hold was lifted. Further details on the investigation into these signals are included in the Supplemental Results, https://links.lww.com/HC9/C451.

### Efficacy

No participant in any of the groups had a ≥1-log decrease in qHBsAg or HBsAg loss at day 337 (Supplemental Table S2, https://links.lww.com/HC9/C451) or any preceding timepoint compared with day 1. No differences in qHBsAg GMCs were observed between the vaccine and control groups, and none of the vaccine groups showed a ≥1-log difference in GMCs at day 337 compared with the GMCs in their respective control (Supplemental Figure S1, https://links.lww.com/HC9/C451, and Supplemental Table S3, https://links.lww.com/HC9/C451). Hence, the efficacy proof-of-principle was not achieved.

### Immunogenicity

**FIGURE 4:**
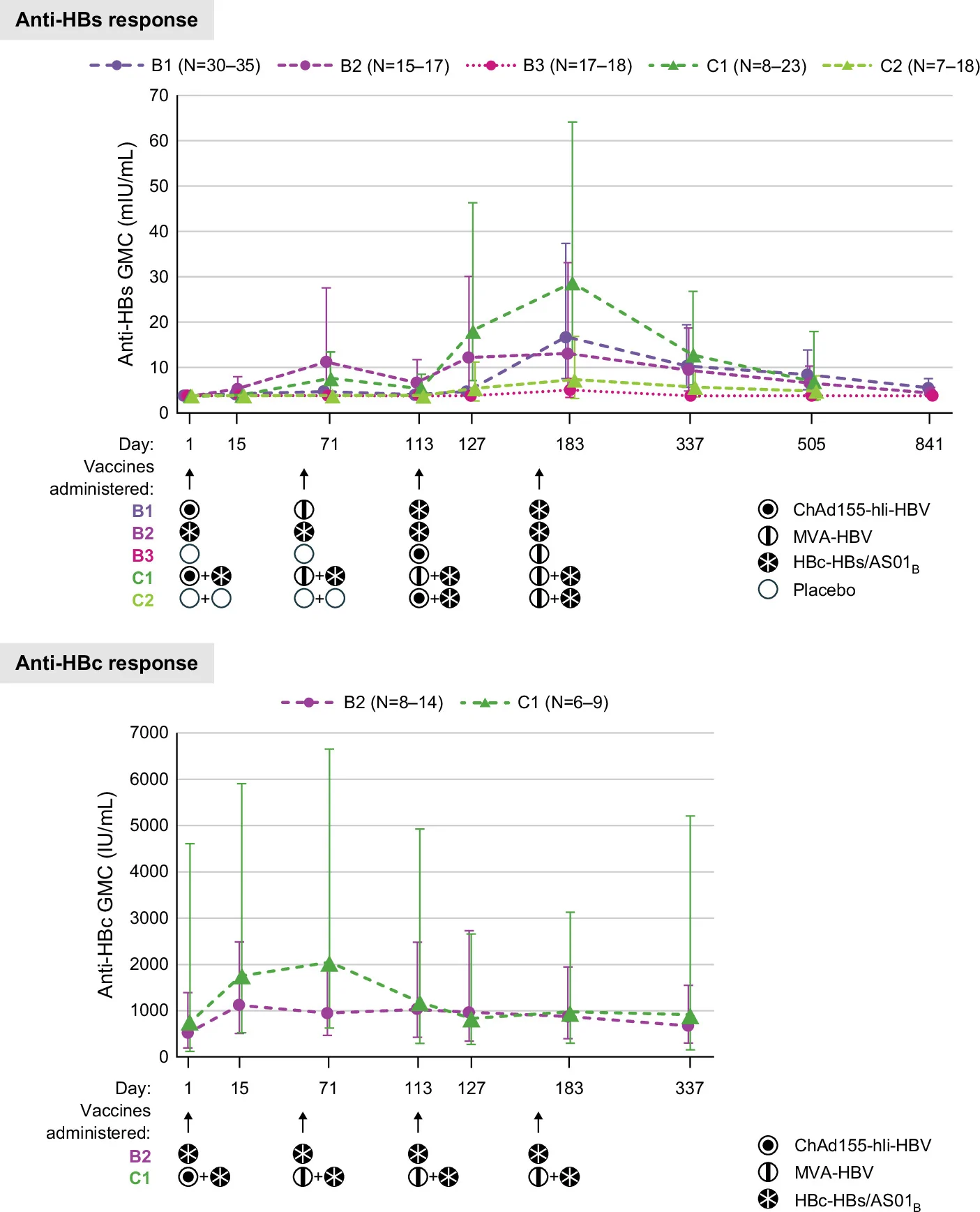
Anti-HBs and anti-HBc geometric mean concentrations in participants from Steps B and C^a^ (modified per-protocol set for immunogenicity). Anti-HBc responses were only assessed in a subset of participants in the B2 and C1 groups. Error bars depict 95% confidence intervals. Interventions administered: B1, high doses of ChAd155-hIi-HBV (prime) on day 1, MVA-HBV (boost) on day 57, and HBc-HBs/AS01_B_ on days 113 and 169; B2, 4 high doses of HBc-HBs/AS01_B_ (days 1, 57, 113, and 169); B3, 2 placebo doses (days 1 and 57), followed by high doses of ChAd155-hIi-HBV (prime) on day 113 and MVA-HBV (boost) on day 169; C1, high dose of ChAd155-hIi-HBV (prime) on day 1 followed by 3 high doses of MVA-HBV (boost) on days 57, 113, and 169, with all 4 doses co-administered with high doses of HBc-HBs/AS01_B_; C2, 2 placebo doses (days 1 and 57), followed by high doses of ChAd155-hIi-HBV (prime) on day 113 and MVA-HBV (boost) on day 169, both co-administered with high doses of HBc-HBs/AS01_B_. GMC, geometric mean concentration; N, number of participants with available data (differed by timepoint). ^a^Results for the groups in Step A are not shown here because group sizes are too small to provide meaningful results.

Co-administration of the viral vectors and adjuvanted proteins (C1 group) induced the strongest anti-HBs response, with 73.7% of participants achieving an anti-HBs concentration ≥10 mIU/mL at 2 weeks post-dose 4 (day 183) (Table 3). Anti-HBs GMCs increased after each subsequent immunization in this group, suggesting a boosting effect of each MVA-HBV and HBc-HBs/AS01_B_ co-administration (Figure 4). Sequential immunization with vectors and proteins in the B1 group resulted in 40.0% of participants reaching anti-HBs ≥10 mIU/mL at 2 weeks post-dose 4 (Table 3, Figure 4). In participants receiving 4 doses of adjuvanted proteins (B2 group), anti-HBs concentrations increased after subsequent doses, with 37.5% achieving anti-HBs concentrations ≥10 mIU/mL at 2 weeks post-dose 4 (Table 3 and Figure 4).

**TABLE 3:** Number and percentage of participants in Steps B and C^a^ with anti-HBs antibody concentration ≥10 mIU/mL (modified per-protocol set for immunogenicity)

	Step B (sequential administration of high doses)						Step C (co-administration of high doses)			
	B1		B2		B3		C1		C2	
Timepoint	n/N	% (95% CI)	n/N	% (95% CI)	n/N	% (95% CI)	n/N	% (95% CI)	n/N	% (95% CI)
Day 1 (pre-dose 1)	0/35	0.0 (0.0–10.0)	0/17	0.0 (0.0–19.5)	0/18	0.0 (0.0–18.5)	0/23	0.0 (0.0–14.8)	0/18	0.0 (0.0–18.5)
Day 15 (2W post-dose 1)	1/34	2.9 (0.1–15.3)	2/17	11.8 (1.5–36.4)	0/18	0.0 (0.0–18.5)	0/23	0.0 (0.0–14.8)	0/18	0.0 (0.0–18.5)
Day 71 (2W post-dose 2)	4/35	11.4 (3.2–26.7)	5/16	31.3 (11.0–58.7)	0/18	0.0 (0.0–18.5)	6/21	28.6 (11.3–52.2)	0/17	0.0 (0.0–19.5)
Day 113 (pre-dose 3)	1/32	3.1 (0.1–16.2)	4/16	25.0 (7.3–52.4)	0/18	0.0 (0.0–18.5)	2/20	10.0 (1.2–31.7)	0/15	0.0 (0.0–21.8)
Day 127 (2W post-dose 3)	4/32	12.5 (3.5–29.0)	6/15	40.0 (16.3–67.7)	0/17	0.0 (0.0–19.5)	9/19	47.4 (24.4–71.1)	1/15	6.7 (0.2–31.9)
Day 183 (2W post-dose 4)	12/30	40.0 (22.7–59.4)	6/16	37.5 (15.2–64.6)	2/18	11.1 (1.4–34.7)	14/19	73.7 (48.8–90.9)	3/13	23.1 (5.0–53.8)
Day 337 (24W post-dose 4)	9/32	28.1 (13.7–46.7)	6/16	37.5 (15.2–64.6)	0/18	0.0 (0.0–18.5)	7/20	35.0 (15.4–59.2)	3/14	21.4 (4.7–50.8)
Day 505 (48W post-dose 4)	9/32	28.1 (13.7–46.7)	4/16	25.0 (7.3–52.4)	0/18	0.0 (0.0–18.5)	2/8	25.0 (3.2–65.1)	1/7	14.3 (0.4–57.9)
Day 841 (96W post-dose 4)	5/30	16.7 (5.6–34.7)	1/15	6.7 (0.2–31.9)	0/18	0.0 (0.0–18.5)	0/0		0/0	

Interventions administered: B1, high doses of ChAd155-hIi-HBV (prime) on day 1, MVA-HBV (boost) on day 57, and HBc-HBs/AS01_B_ on days 113 and 169; B2, 4 high doses of HBc-HBs/AS01_B_ (days 1, 57, 113, and 169); B3, 2 placebo doses (days 1 and 57), followed by high doses of ChAd155-hIi-HBV (prime) on day 113 and MVA-HBV (boost) on day 169; C1, a high dose of ChAd155-hIi-HBV (prime) on day 1 followed by 3 high doses of MVA-HBV (boost) on days 57, 113, and 169, with all 4 doses co-administered with high doses of HBc-HBs/AS01_B_; C2, 2 placebo doses (days 1 and 57), followed by high doses of ChAd155-hIi-HBV (prime) on day 113 and MVA-HBV (boost) on day 169, both co-administered with high doses of HBc-HBs/AS01_B_. n/%, number/percentage of participants with anti-HBs antibody concentration ≥10 mIU/mL; N, number of participants with available results; W, weeks.

^a^ Results for the groups in Step A are not shown here because group sizes are too small to provide meaningful results.

All participants who were tested for anti-HBc (ie, subset of B2 and C1) had detectable antibody concentrations at all timepoints. Anti-HBc GMCs tended to be highest at 2 weeks post-dose 1 (day 15) in B2 and 2 weeks post-dose 2 (day 71) in C1. At these timepoints, GMCs tended to be higher in C1 (co-administration of viral vectors and adjuvanted proteins) than in B2 (administration of adjuvanted proteins alone), but no further increase in GMCs was observed after dose 3 or dose 4 in either group (Figure 4).

**FIGURE 5:**
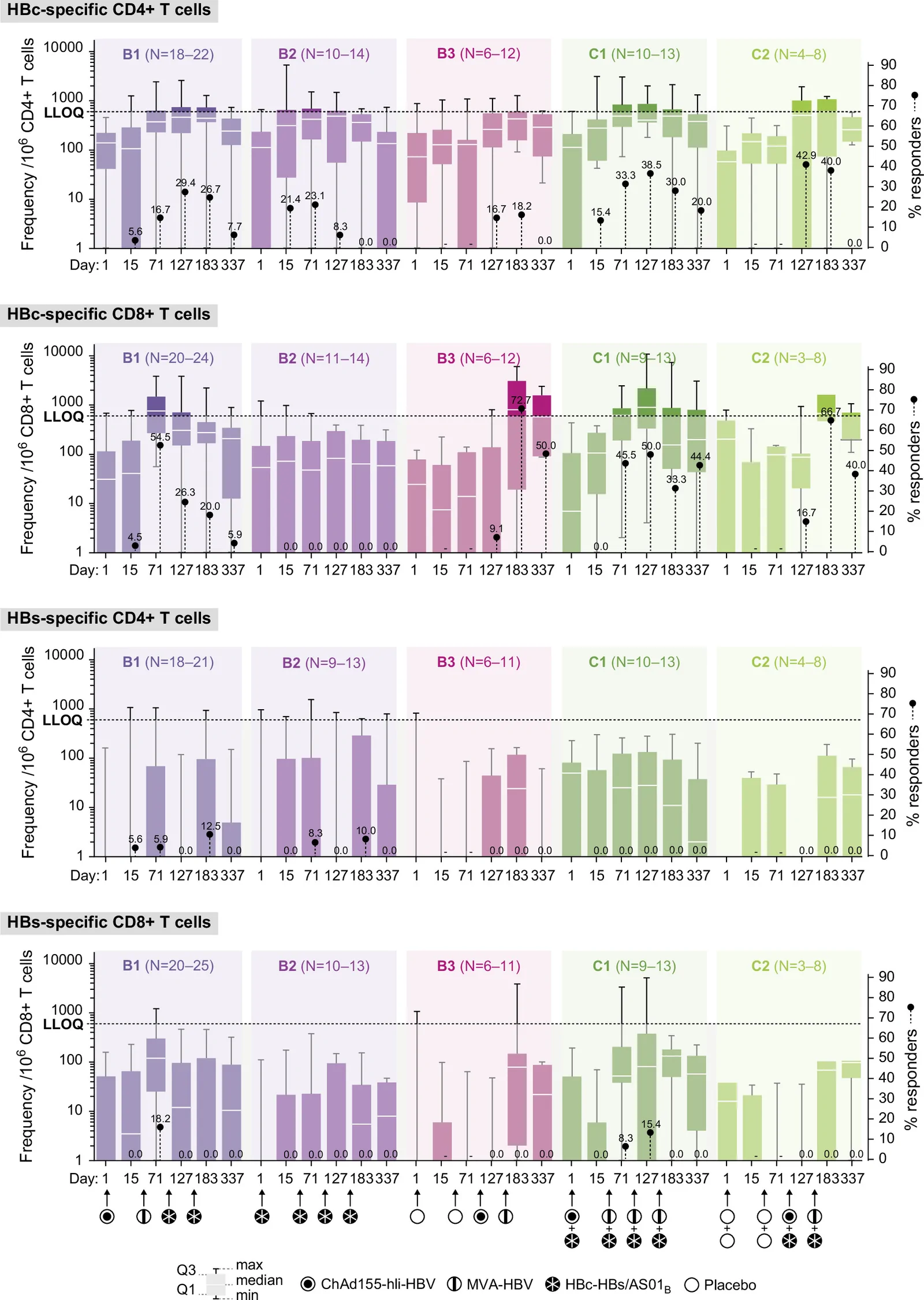
HBc-specific and HBs-specific polypositive CD4+ and CD8+ T-cell frequencies and responders in participants from Steps B and C^a^ (modified per-protocol set for immunogenicity). A responder is defined as a participant with a post-vaccination/pre-vaccination ratio in the treatment group above the maximum post-vaccination/pre-vaccination ratio observed in the respective placebo controls (B3 or C2) over the timepoints up to day 113; the maximum ratio in B3 was 1.24 for HBc CD4+ and 1.00 for HBc CD8+, HBs CD4+, and HBs CD8+; the maximum ratio in C2 was 1.08 for HBc CD4+ and 1.00 for HBc CD8+, HBs CD4+, and HBs CD8+. Interventions administered: B1, high doses of ChAd155-hIi-HBV (prime) on day 1, MVA-HBV (boost) on day 57, and HBc-HBs/AS01_B_ on days 113 and 169; B2, 4 high doses of HBc-HBs/AS01_B_ (days 1, 57, 113, and 169); B3, 2 placebo doses (days 1 and 57), followed by high doses of ChAd155-hIi-HBV (prime) on day 113 and MVA-HBV (boost) on day 169; C1, high dose of ChAd155-hIi-HBV (prime) on day 1 followed by 3 high doses of MVA-HBV (boost) on days 57, 113, and 169, with all 4 doses co-administered with high doses of HBc-HBs/AS01_B_; C2, 2 placebo doses (days 1 and 57), followed by high doses of ChAd155-hIi-HBV (prime) on day 113 and MVA-HBV (boost) on day 169, both co-administered with high doses of HBc-HBs/AS01_B_. LLOQ, lower limit of quantification (590 HBs-specific or HBc-specific polypositive CD4+ or CD8+ T cells per 10^6^ CD4+ or CD8+ T cells); N, number of participants with available data (differed by timepoint). ^a^Results for the groups in Step A are not shown here because group sizes are too small to provide meaningful results.

Both sequential and concomitant administration of the vectors and adjuvanted proteins (B1 and C1, respectively) induced HBc-specific polypositive T-cell responses in some of the participants. The low HBc-specific CD4+ T-cell responses elicited by dose 1 (ChAd155-hIi-HBV prime) were enhanced by dose 2 (MVA-HBV boost) and remained stable after doses 3 and 4 in both groups, with 26.7% of CD4+ T-cell responders in B1 and 30.0% in C1 at 2 weeks post-dose 4 (Figure 5). HBc-specific CD8+ T-cell frequencies were boosted by dose 2 and declined post-dose 3 (in B1) or post-dose 4 (in C1). The percentages of responders were 54.5% in B1 and 45.5% in C1 at 2 weeks post-dose 2, and 20.0% and 33.3%, respectively, at 2 weeks post-dose 4 (Figure 5). Among participants receiving 4 doses of adjuvanted proteins (B2), a mild HBc-specific CD4+ but no CD8+ T-cell response was observed (Figure 5). Limited or no HBs-specific CD4+ and CD8+ T-cell responses were observed in all groups (Figure 5).

### ChAd155 vector shedding

No ChAd155 vector DNA was detected in throat swabs or urine samples of any of the participants in Step B who received either ChAd155-hIi-HBV, control vaccine, or placebo at any of the timepoints up to day 31.

## DISCUSSION

This first-in-human study showed that an immunization strategy relying on a heterologous prime-boost approach with the viral vector vaccines ChAd155-hIi-HBV and MVA-HBV, combined with sequential or concomitant administration of an AS01_B_-adjuvanted HBc-HBs protein vaccine, had an acceptable safety profile in chronic hepatitis B patients controlled on NA therapy. The different schedules induced humoral and cell-mediated immune responses, but this did not translate into the anticipated efficacy outcome.

Both viral vector vaccines and the adjuvanted proteins were more reactogenic than the placebo. Most reactions were mild or moderate, and severe (grade 3) reactions did not last longer than 4 days. A higher proportion of participants reported grade 3 solicited AEs after the co-administration regimen compared with the sequential regimens. This difference was predominantly observed after dose 2 and may reflect random variation. No increase in the incidence of solicited AEs was observed with subsequent doses. This study was also the first to evaluate the safety of 4 doses of an AS01-adjuvanted vaccine (B2 group); the findings indicate that 4 doses had an acceptable safety profile. Two safety signals (cardiac events and hematuria and proteinuria events) were refuted after an in-depth investigation by the sponsor’s safety review team. No SAEs deemed related to immunization were reported, none of the participants withdrew from the study due to an AE, and no new safety concerns were identified, supporting the acceptable safety profile of the different immunization regimens. No ChAd155 vector DNA was detected in urine or saliva after immunization with ChAd155-hIi-HBV, confirming that the ChAd155 vector was replication-deficient, supporting its safety profile.

Several of the therapeutic vaccine regimens evaluated in our study induced HBV-specific antibody and T-cell responses. However, no participant experienced a ≥1-log reduction in or loss of HBsAg by 24 weeks post-dose 4. This indicates that none of the tested regimens conferred a functional cure. This is in line with other studies showing that therapeutic vaccines as a monotherapy fail to reduce HBsAg levels in chronic hepatitis B patients who are not selected based on baseline HBsAg or immune status, even though these vaccines can induce HBV-specific immune responses.^16^,^24^,^25^,^26^ This lack of efficacy seems to be independent of the vaccine platform and suggests that the immune response induced by therapeutic vaccines may not be potent enough to overcome immune exhaustion and achieve sustained HBsAg loss.^16^ Several other candidate therapeutic vaccines typically elicited only limited T-cell responses in persons with chronic hepatitis B.^16^ In addition, in a previous trial evaluating a chimpanzee adenoviral and MVA vector prime-boost approach, reductions in HBsAg levels among participants who received the viral vectors alone were observed in only a small number of patients with low baseline HBsAg (<50 IU/mL).^25^ In our study, only patients with an HBsAg concentration >50 IU/mL were enrolled; very few had HBsAg between 50 and 100 IU/mL, and more than two-thirds of the participants had HBsAg levels ≥1000 IU/mL at screening. The limited number of patients with low baseline HBsAg may have further hampered the ability to observe vaccine efficacy because HBsAg is a key mediator of immune tolerization. Therapeutic vaccines likely require combination therapy for success.^14^,^27^ For example, pretreatment with agents that reduce HBsAg before therapeutic immunization and/or concurrent treatment with checkpoint inhibitors or other immune-activating agents might improve efficacy.^16^ Several trials are currently ongoing that evaluate combination therapies of therapeutic vaccines with a variety of other agents.^14^,^27^

We observed a synergistic effect of co-administering viral vectors with adjuvanted proteins on the induction of anti-HBs antibody responses, with a boosting effect of each co-administration. Both sequential and concomitant administration of vectors and proteins induced HBc-specific CD4+ and CD8+ T cells, but only limited or no HBs-specific T cells (likely due to immune tolerance resulting from high circulating HBsAg levels). Administration of MVA-HBV boosted the HBc-specific CD8+ T-cell response elicited by ChAd155-hIi-HBV, highlighting the benefits of the heterologous prime-boost strategy, corroborating previous observations with other antigens.^19^,^20^,^21^ As expected based on findings with other AS01-adjuvanted vaccines,^23^,^28^ a mild increase in the frequencies of HBc-specific CD4+ T cells but not of CD8+ T cells was observed after immunization with adjuvanted proteins. No boosting effect of subsequent doses of adjuvanted proteins was observed.

Strengths of our study include that it evaluated efficacy, immunogenicity, and safety in the same population, and that it examined 2 dose levels and different schedules, with several active controls. Our study has several limitations. Group sample sizes were small, and all endpoints were exploratory with no adjustment for multiplicity, warranting caution when interpreting the results. In addition, because the study was terminated early, some immunoassays were only performed on a subset of participants, further reducing group sizes, particularly for the later timepoints and the Step C groups. Anti-HBc assessments were performed in a small number of participants in only 2 groups (B2 and C1) and not in the groups receiving a placebo, limiting interpretation of the results. Our study excluded patients with HBsAg <50 IU/mL; this may have hampered the ability to see reductions in HBsAg, as previous research indicated that a functional cure may be easier to achieve in patients with low levels of HBsAg.^25^ Finally, our study exclusively evaluated immunogenicity in peripheral blood and did not assess intrahepatic immune responses. Therefore, we could not determine whether the observed peripheral immune activation translated into effective immune activity within the liver, which is likely critical for achieving clinical efficacy.

In summary, therapeutic immunization with ChAd155-hIi-HBV and MVA-HBV, followed by or co-administered with HBc-HBs/AS01_B_, had an acceptable safety profile in patients with chronic hepatitis B receiving NA therapy. However, due to a lack of efficacy, clinical development of this approach as a monotherapy was terminated. Future studies are needed to determine whether combining any of these components with other treatment strategies can achieve a functional cure in patients with chronic hepatitis B.

## Supplementary Material
