# Supplementary material for: A randomized trial of viral vector and adjuvanted protein HBV therapeutic vaccine in people with chronic hepatitis B on nucleos(t)ide analogs
Source: Hepatol Commun. 2026 Aug 20;10(9):e01025. doi: 10.1097/HC9.0000000000001025 (PMC13496306; doi:10.1097/HC9.0000000000001025)
Supplement: Supplementary file 1 [file hc9-10-e01025-s001.docx]

**Manuscript ID: HEP4-26-0435**

**Supplementary material**

**Phase 1/2 randomized trial of a therapeutic vaccine with heterologous prime-boost viral vectors and HBV core and surface proteins in people with chronic hepatitis B on nucleos(t)ide analogs**

Wen-Juei Jeng et al.

**Plain language summary**

**What is the context?**

- Hepatitis B is a serious liver infection caused by the hepatitis B virus.
- In some people, hepatitis B can become chronic, which increases the risk of liver scarring, liver failure, and liver cancer.
- In patients with chronic hepatitis B, the immune system is dysfunctional and can no longer fight the infection as effectively.
- Most people with chronic hepatitis B receiving nucleos(t)ide analogs will need lifelong antiviral treatment.

**What is new?**

- This study evaluated an investigational therapeutic vaccination strategy that aimed to restore the immune system’s ability to fight the hepatitis B virus.
- The strategy consisted of injecting different types of therapeutic vaccines (two different viral vectors and adjuvanted proteins), either one after another or multiple injections of vector and proteins together. The patients included in the study were 18–65 years of age and had chronic hepatitis B that was controlled with antiviral medication.
- Severe reactions after vaccination typically resolved within 2–3 days, and no safety concerns were identified.
- The vaccines induced a modest immune response, which was strongest when they were injected together.
- However, the vaccines were not effective in lowering the levels of the viral antigen HBsAg, meaning they would not be able to achieve long-lasting virus control.

**What is the impact?**

- The tested therapeutic vaccination strategies were not effective enough to achieve the desired results of lowering the levels of viral antigen in patients with chronic hepatitis B controlled with antiviral medication.

Further research is needed to find more effective treatments for patients with chronic hepatitis B.

**Supplementary methods**

**Inclusion and exclusion criteria**

***Inclusion criteria***

- Patients who, in the opinion of the investigator, could and would comply with the requirements of the protocol (e.g., completion of the diary cards, return for follow-up visits).
- Written informed consent obtained from the patient prior to performing any study-specific procedure.
- A male or female between, and including, 18 and 65 years of age at the time of the first immunization.
- Female patients of non-childbearing potential could be enrolled in the study. Non-childbearing potential was defined as hysterectomy, bilateral ovariectomy, or post-menopause.
- Female patients of childbearing potential could be enrolled in the study if the patient:
  - has practiced adequate contraception for 30 days prior to immunization, and
  - had a negative pregnancy test at screening, and
  - had agreed to continue adequate contraception from screening until 12 weeks after completion of the immunization series.
- Male patients could be enrolled in the study if the patient:
  - had documented bilateral vasectomy and resultant azoospermia, bilateral orchiectomy or azoospermia, or
  - had agreed to practice abstinence from penile-vaginal intercourse (if this was their preferred and usual lifestyle) or use condoms from screening until 12 weeks after completion of the vaccination series.
- Patient with chronic hepatitis B, under and adherent to treatment with a nucleos(t)ide analog with high barrier to resistance (e.g., entecavir, tenofovir disoproxil fumarate, tenofovir alafenamide) given as per approved label/dosage for at least 24 months.
- Documented medical history of HBeAg-negative chronic hepatitis B prior to onset of nucleos(t)ide analog therapy (applicable to all patients in Step A and Step B and to some patients in Step C) or documented medical history of HBeAg-negative chronic hepatitis B over a period of at least 24 months prior to screening (applicable to some patients in Step C only).
- Documented HBV suppression as per local clinical diagnosis within the previous 24 months and at screening test HBV DNA <10 IU/mL. If no results were available, two screening tests needed to be performed at least 2 weeks apart. Small fluctuations of HBV DNA (≤10x lower limit of quantification [LLOQ], with LLOQ defined by the laboratory that performed the testing) were allowed if HBV DNA was <10 IU/mL at screening and was clearly not rising during the previous 24 months.
- Documented normal level of ALT as per local clinical diagnosis within the previous 24 months and ALT ≤48 U/L at screening test. Small fluctuations of ALT (≤1.5x upper limit of normal [ULN]) were allowed if ALT was ≤48 U/L at screening. If no results were available, two screening tests needed to be performed at least 2 weeks apart. ULN was defined according to the local laboratory reference range.
- No clinical diagnosis of cirrhosis (e.g., F4 by METAVIR scoring system, or ≥6 by Ishak scoring system, or FibroScan transient elastography (TE) score >12.5 kPa) within the previous 24 months.
- FibroScan TE score <9.6 kPa and FibroTest score <0.59 at screening. A patient with one of these parameters out of range, but with a liver biopsy within 12 months before screening that showed F0–2 by METAVIR scoring system or stage 0–4 by Ishak scoring system, could be included.
- HBsAg concentration >50 IU/mL and anti-HBs-negative at screening.
- Anti-HBc-positive at screening.
- HBeAg-negative at screening.

*Additional inclusion criteria for enrollment in the ancillary study*

- Patients who, in the opinion of the investigator, could and would comply with the requirements of the protocol.
- Written informed consent obtained from the patient prior to performance of any study-specific procedure.
- Patients who met all inclusion criteria and were randomized to Step B of the main study.

***Exclusion criteria***

- Use of any investigational or non-registered product (drug or vaccine) other than the study vaccines during the period starting 30 days before the first dose of study vaccines (day ˗29 to day 1), or planned use during the study period.
- Any medical condition that in the judgment of the investigator would have made intramuscular injection unsafe.
- Chronic administration (defined as more than 14 days in total) of immunosuppressants or other immune-modifying drugs (including but not limited to interferon) during the period starting 6 months prior to the first vaccine dose. For corticosteroids, this meant prednisone ≥10 mg/day or equivalent. Inhaled and topical steroids were allowed.
- Administration of immunoglobulins and/or any blood products during the period starting 3 months before the first dose of study vaccines or planned administration during the study period.
- Use of systemic cytotoxic agents, chronic antiviral agents or Chinese herbal medicines that, in the opinion of the investigator, could have activity against HBV, within the previous 6 months prior to randomization into this study. Antiviral treatment/prevention for influenza or herpes simplex virus was allowed.
- Administration of adenovirus/adenovector-based or modified vaccinia Ankara (MVA)-based vaccine within the last 12 months except for adenovirus/adenovector-based COVID-19 vaccines, which could be administered up to 30 days prior to the first study vaccine dose (applicable for all patients except for the patients in France) or administration of adenovirus/adenovector-based or MVA-based vaccine within the last 12 months (applicable for the patients in France only).
- (Planned) administration of a vaccine not foreseen by the study protocol in the period starting 14 days before each dose and ending 30 days after each dose of vaccines, with the exception of influenza vaccine, which could be given at any time except within a 7-day period before or after each vaccine dose, and COVID-19 vaccine, which could be given at any time except within a 30-day period before or after each vaccine dose. COVID-19 mRNA based-vaccines could be administered any time except for the period of 14 days before and 30 days after each study vaccine dose. If the type of COVID-19 vaccine was unknown, the allowed interval of 30 days before or after each study vaccine dose had to be followed.
- Treatment with nephrotoxic drugs (e.g., aminoglycosides, amphotericin B, vancomycin, cidofovir, foscarnet, cis-platinum, pentamidine) or competitors of renal excretion (e.g., probenecid) within 2 months prior to screening or the expectation that the patient would receive any of these during the course of the study. Tenofovir alafenamide/tenofovir disoproxil fumarate given as nucleos(t)ide analog therapy was allowed.
- Concurrently participating in another clinical study, at any time during the study period, in which the patient had been or would be exposed to an investigational or a non-investigational vaccine/product (pharmaceutical product or device).
- Medical history of cirrhosis.
- Medical history of hepatic decompensation (e.g., ascites, spontaneous bacterial peritonitis, hepatorenal syndrome, variceal bleeding, or hepatic encephalopathy).
- Planned for liver transplantation or previous liver transplantation.
- Personal or family (first degree) history of autoimmune disease.
- Family history of congenital or hereditary immunodeficiency.
- History of any reaction or hypersensitivity likely to be exacerbated by any component of the vaccines.
- Evidence of HCV or HDV infection. History of acute hepatitis A or acute hepatitis E was not an exclusion criterion.
- Suspicion of or confirmed hepatocellular carcinoma or any other liver cancer in medical history or at screening:
  - Suspicious foci at liver imaging exam.
  - Elevated α-fetoprotein >50 ng/mL.
- Documented evidence of other currently active cause of hepatitis (e.g., autoimmune hepatitis, primary biliary cirrhosis; primary sclerosing cholangitis, hemochromatosis, alpha-1 antitrypsin deficiency, Wilson's disease).
- Hematology and biochemistry parameters outside normal clinical range at screening:
  - Biochemistry:
    - Glomerular filtration rate <60 mL/min
    - Bilirubin >27.5 μmol/L unless it was considered as clinically not significant by the investigator or the diagnosis of Gilbert Syndrome had been established and confirmed by the investigator
    - Gamma-glutamyl transferase >65 U/L for males or >45 U/L for females unless it was considered as clinically not significant by the investigator
    - ALT >48 U/L
    - AST >42 U/L unless it was considered as clinically not significant by the investigator
    - Alkaline phosphatase >125 U/L unless it was considered as clinically not significant by the investigator
  - Hematology:
    - Hemoglobin <12.0 g/dL (for females) or <13.5 g/dL (for males) unless it was considered as clinically not significant by the investigator
    - Red blood cell count <3.9×10^6^ cells/mm^3^ (females) or <4.4×10^6^ cells/mm^3^ (males) unless it was considered as clinically not significant by the investigator
    - White blood cell count <3500 cells/mm^3^ or >12,000 cells/mm^3^ unless it was considered as clinically not significant by the investigator
    - Platelets <140,000 cells/mm^3^
    - International normalized ratio >1.32 (i.e., 1.1×ULN)
- Known diabetes type I.
- Body mass index >35 kg/m² at screening.
- Any serious or active medical or psychiatric illnesses other than chronic hepatitis B, which, in the opinion of the investigator, would interfere with patient treatment, assessment or compliance with the protocol. This included any uncontrolled clinically significant renal, cardiac, pulmonary, vascular, neurogenic, digestive, metabolic (diabetes, thyroid disorders, adrenal disease), immunodeficiency disorders, or cancer.
- History of or current drug abuse and/or excess of alcohol consumption as defined per local guidelines.
- HIV-positive patient.
- Pregnant or lactating female.
- Female planning to become pregnant or planning to discontinue contraceptive precautions in the period starting from screening up to 12 weeks post-last immunization visit.
- Fever and/or acute minor illness (such as mild diarrhea, mild upper respiratory infection) could be enrolled for screening at the discretion of the investigator, provided that the condition was resolved at the time of immunization.

**Staggered design and dose escalation**

Within each Step, maximum two participants per group were initially randomized, immunized, and followed for 2 days. After these participants had completed visit 2 (2 days post-dose 1), an internal safety review committee (SRC, composed of a safety physician, a hepatologist, a clinical research and development lead, and a biostatistician who were not otherwise involved in therapeutic HBV studies) reviewed all available safety data and decided whether to continue with the randomization and immunization of the remaining participants in each group. If two or more participants were randomized at the same study site on the same day, they were to be immunized sequentially at least 60 minutes apart to allow monitoring of any acute event (e.g., anaphylactic reaction). Administration of subsequent doses (doses 2, 3, and 4) to the first participants in each group could only be done if a favorable evaluation from the SRC was available of safety data up to 30 days after doses 1, 2, and 3, respectively.

In terms of dose escalation, when all participants from Step A (low doses of vectors and proteins, administered sequentially, planned N=15) had completed visit 8 (14 days post-dose 2), the SRC reviewed all available safety data and decided whether it was appropriate to continue the study and start Step B randomization and immunization. Safety assessment of at least 12 patients in Step A with available data up to visit 8 could be considered sufficient for moving to Step B, if approved by local authorities. In Step B (high doses of vectors and proteins, administered sequentially), approximately 40 participants were randomized and immunized first. When they had completed visit 8, the SRC reviewed all available safety data and decided whether to start randomization and immunization of the remaining Step B participants. After Step B enrollment was complete, Step C randomization and immunization (high doses of vectors and proteins, co-administered) could start. Before initiating the randomization for each Step, the study team reviewed the number of participants available for that step.

**Vaccines**

The ChAd155-hIi-HBV investigational vaccine consists of a recombinant replication-deficient chimpanzee-derived adenovirus group C serotype 155 vector (ChAd155) encoding a fusion of sequences derived from the truncated nucleocapsid core protein antigen (HBcAg) and the full-length surface antigen (HBsAg), separated by the self-cleaving 2A region of the foot-and-mouth disease virus that allows processing of the fusion protein into separate protein antigens. The N-terminal part of the HBcAg gene was fused to the gene encoding the human MHC class II-associated invariant chain p35 isoform (hIi).

The MVA-HBV investigational vaccine consists of the replication-deficient orthopoxvirus modified vaccinia Ankara recombinant vector, which contains a transgene encoding truncated HBcAg and full-length HBsAg, separated by the foot-and-mouth disease virus self-cleaving 2A region.

The HBc-HBs/AS01_B_ investigational vaccine contains AS01_B_ adjuvant and a recombinant protein lyophilized vaccine mix containing the truncated HBcAg (produced using *Escherichia coli*) and full-length HBsAg (produced using *Saccharomyces cerevisiae*). The recombinant HBcAg is derived from HBV genotype A and is C-terminally truncated to remove the RNA-binding domain. The recombinant HBsAg, also derived from HBV genotype A, is the same protein present in several licensed HBV vaccines (e.g., *Engerix*, GSK). AS01_B_ contains 50 µg 3-*O*-desacyl-4′-monophosphoryl lipid A, 50 µg *Quillaja saponaria* Molina, fraction 21 (licensed by GSK from Antigenics LLC, a wholly owned subsidiary of Agenus Inc., a Delaware, USA corporation), and liposome.

**Study holding rules**

The study was overseen by an internal SRC, operating under a charter. They determined whether any of the predefined study holding rules were met or whether there were any other safety signals. If the SRC determined that a holding rule was met, the study would be put on hold. All immunizations would cease immediately, but all other procedures relating to safety, immunology, efficacy, and disease monitoring would continue. Following an internal review, the sponsor would then decide to suspend, modify, or continue the conduct of the study. Details on study holding rules are provided in the study protocol, which is available on <https://www.gsk-studyregister.com/trials/204852>.

**List of ethics committees**

***Belgium***

Main study: Institutional Review Board - ZNA/OCMW Antwerpen, Commissie Medische Ethiek - ZNA Middelheim, Antwerpen; Ethics Committee of Cliniques Universitaires Saint-Luc, Brussels; Ethics Committee of Hôpital Erasme, Gastro-entérologie, Brussels; CHU Saint-Pierre, Comité d'Ethique, Brussels; Universitair Ziekenhuis Leuven, Ethisch Comité; Universitair Ziekenhuis Gent, Ethisch Comité; Universitair Ziekenhuis Antwerpen, Comité voor Medische Ethiek

***France***

Main study: Comité de Protection des Personnes Ouest IV, Nantes

Ancillary study: Comité de Protection des Personnes Sud-Ouest et Outre-mer IV, Limoges

***Germany***

Main study: Landesamt fuer Gesundheit und Soziales Berlin, Main office of the Ethics Committee Berlin; Ethics Committee II of the University Heidelberg (Medical Faculty Mannheim); Ethics Committee of the Medical College Hannover; Ethics Committee of the Medical Faculty of University Duisburg-Essen, University Clinic Essen; Main Office of the Ethics Committee of University Clinic Bonn; Ethics Committee of the Internal Affairs and Medical Association Rheinland-Pfalz, Mainz

Ancillary study: Ethik-Kommission der Med. Hochschule Hannover (MHH); Ethik-Kommission der Med. Fakultaet am Universitaetsklinikum Tuebingen; Ethik-Kommission bei der Aerztekammer Hamburg; Ethik-Kommission der Landesaerztekammer Rheinland-Pfalz, Mainz; Ethik Kommission an der Medizinischen Fakultaet der RWTH Aachen

***Hong Kong***

Main study: Institutional Review Board of The University of Hong Kong/Hospital Authority Hong Kong West Cluster, Pokfulam

***Poland***

Main study: Office for Reg. of Med. Products, Medical Devices and Biocidal Products, Department of Clinical Trial on Medicinal Products, Warsaw; Bioethics Committee at the Regional Medical Chamber, Rzeszow; Komisja Bioetyczna przy Uniwersytecie Medycznym w Lublinie, Lublin

***Spain***

Main and ancillary study: CEIm University Hospital Puerta de Hierro-Majadahonda, Majadahonda-Madrid

***Taiwan***

Main and ancillary study: National Cheng Kung University Hospital Institutional Review Board, Tainan; Taipei Veterans General Hospital Institutional Review Board, Taipei; Chang Gung Medical Foundation Institutional Review Board, Taipei; China Medical University Hospital Research Ethics Committee, Taichung City; Taichung Veterans General Hospital Institutional Review Board, Taichung

***Thailand***

Main and ancillary study: Institutional Review Board, Faculty of Medicine, Chulalongkorn University, Bangkok; Research Ethics Committee, Faculty of Medicine, Chiang Mai University, Chiang Mai

***United Kingdom***

Main and ancillary study: South Central - Oxford A Research Ethics Committee, Bristol

**List of objectives and endpoints**

Primary and secondary study objectives and endpoints are provided in the table below.

| **Objective** | **Endpoint** |
| --- | --- |
| **Primary** |  |
| To assess the safety of escalating doses of the viral vector HBV vaccines (prime-boost) and/or adjuvanted protein vaccines in patients with chronic HBV infection virally suppressed on NA therapy | Occurrence of AEs from vaccination up to day 337:  − Occurrence of each solicited administration-site and systemic event within 7 days after each vaccination (from day of vaccination to 6 days after vaccination)  − Occurrence of unsolicited AEs within 30 days after each vaccination (from day of vaccination to 29 days after vaccination)  − Occurrence of hematological, biochemical, or urinalysis laboratory abnormalities within 30 days after each vaccination  − Occurrence of SAEs, pIMDs, liver disease-related AESIs, hematological AESIs, and MAEs up to 6 months after the last dose (day 337) |
| **Secondary** |  |
| To assess the immunogenicity of the viral vector HBV vaccines (prime-boost) and/or adjuvanted protein vaccines in patients with chronic HBV infection virally suppressed on NA therapy | Anti-HBc antibodies: seropositivity and concentration  Anti-HBs antibodies: seroconversion and concentration; anti-HBs ≥10 mIU/mL and  ≥100 mIU/mL  Frequency of HBc- and HBs-specific CD4+ T cells and CD8+ T cells; CD4+ T-cell responders, CD8+ T-cell responders |
| To assess the efficacy of the viral vector HBV vaccines (prime-boost) and/or adjuvanted protein vaccines in patients with chronic HBV infection virally suppressed on NA therapy | qHBsAg: number of patients with ≥0.5 log decrease, ≥1-log decrease, HBsAg loss, and log-changes since pre-vaccination  Number of patients with HBsAg loss and anti-HBs seroconversion  Mean qHBsAg in each group |
| To assess the long-term safety of escalating doses of the viral vector HBV vaccines (prime-boost) and/or adjuvanted protein vaccines in patients with chronic HBV infection virally suppressed on NA therapy | Occurrence of AEs from vaccination up to day 841:  − Occurrence of any SAEs, SAEs causally related to an investigational vaccine, MAEs, pIMDs, liver disease-related AESIs, and hematological AESIs throughout the study period  − Occurrence of AEs and SAEs leading to study withdrawal  Pregnancy and pregnancy outcome throughout the study period |

AE, adverse event; AESI, adverse event of special interest; MAE, medically attended event; NA, nucleos(t)ide analog; pIMD, potential immune-mediated disease; SAE, serious adverse event.

**Liver disease-related adverse events of special interest (AESIs)**

Liver disease-related AESIs were characterized by one or more of the following:

*ALT flares*

- Elevation of ALT >3×ULN:
  - Mild: >3–5×ULN
  - Moderate: >5–10×ULN
  - Severe: >10×ULN

*ALT flares with other substantial biochemical changes*

- Bilirubin ≥2×ULN
- And/or international normalized ratio >1.5

*Hepatic decompensation*

- Occurrence of one or more of the following events: ascites, spontaneous bacterial peritonitis, hepatorenal syndrome, variceal bleeding, or hepatic encephalopathy

*HBV DNA breakthrough*

- Any increase in serum HBV DNA by >1 log_10_ from nadir or redetection of serum HBV DNA at levels 10-fold the LLOQ of the viral load after HBV DNA was undetectable

In case of liver disease-related AEs, the participants were queried for possible medication/herbal substances that may have altered these parameters, as well as for the following signs/symptoms: appearance or worsening of fatigue, nausea, vomiting, abdominal pain, jaundice, and fever.

**Thrombosis with thrombocytopenia syndrome (TTS)**

Individuals diagnosed with thrombocytopenia during the study had to be actively investigated for signs of thrombosis. Similarly, individuals who presented with thrombosis during the study had to be evaluated for thrombocytopenia. For diagnostic algorithm and clinical management of TTS, clinical guidelines and local recommendations had to be followed. The Brighton Collaboration case definition of TTS was used.

**Laboratory assays**

***Anti-HBc enzyme-linked immunosorbent assay***

A quantitative total anti-HBc assay was developed at GSK using the Siemens Atellica IM HBcT2 commercial kit. This commercial kit is a qualitative assay and is based on a sandwich design to detect total immunoglobulins (Ig) against the HBc protein in human serum. The testing is performed by the Atellica automaton, according to manufacturer’s recommendations. Briefly, serum samples are pretreated with a surfactant to minimize non-specific binding, followed by the addition of streptavidin-coated paramagnetic microparticles coated with biotinylated recombinant HBcAg. If anti-HBc antibodies are present, an antibody-antigen complex forms. Non-magnetic microparticles are incorporated to enhance specificity by reducing false positives. A detection mixture containing acridinium ester-labeled mouse monoclonal anti-human IgG Fab fragments and recombinant HBcAg is added to improve sensitivity. The resulting immune complexes undergo chemiluminescent detection, with relative light units (RLUs) directly proportional to the anti-HBc antibody concentrations in the sample. To transform this qualitative assay into a quantitative format, RLUs are calibrated against a standard curve calibrated against the international reference (WHO 95/522), tested in parallel to the samples. A four-parameter logistic algorithm is used for the standard curve fitting, enabling determination of total anti-HBc antibody concentrations from RLU.

***Cytokine flow cytometry***

Blood samples were collected by venipuncture and peripheral blood mononuclear cells (PBMCs) prepared by centrifugation onto a *Lymphoprep* cushion within 24 hours following collection before storage in liquid nitrogen until testing. To measure T-cell responses elicited by the vaccine candidates (assay performed in GSK Belgium laboratory), PBMCs were stimulated 2 hours using either culture medium (to evaluate the non-specific background response) or a pool of overlapping peptides (15mers overlapping by 11) covering the entire sequence of the HBcAg or HBsAg vaccine antigens. Next, Brefeldin A was added after 2 hours to inhibit cytokine secretion during an overnight (~18 hours) incubation. Cells were then harvested, stained for surface markers (CD4 and CD8), and fixed. Next, the fixed cells were permeabilized, stained with antibodies specific to CD3 and to the assessed activation markers (CD40 ligand, 4-1BB, interferon γ, tumor necrosis factor α, and interleukin-2, -13, and -17), washed, and analyzed by flow cytometry. The results of the intracellular cytokine assays were expressed (after background subtraction) as the frequency of antigen-specific CD4+ or CD8+ T cells (expressing at least two activation markers, including at least one cytokine) per million total CD4+ or CD8+ T cells.

The gating strategy for the ICS-10p assay analyzed using FlowJo software included the following steps, as shown previously^1^:

1. A time gate was created to ensure that the events were captured once the flow stream had stabilized (side scatter-area [SSC-A] versus time density plot)
2. A gate was made on singlets (doublets were excluded [forward scatter-height (FSC-H) versus FSC-A plot])
3. A gate was made on live cells (dead cells were excluded [live-dead dye parameter versus SSC-A plot])
4. A lymphocyte gate was created based on size and granularity (FSC-H [size] versus SSC-A [granularity] plot)
5. CD3+ T cells were gated on (CD3 versus interferon γ plot; as the CD3 marker expression is downregulated after stimulation, a polygon gate was used)
6. CD4+ (CD4+CD8-) and CD8+ (CD8+CD4-) T cells were then gated on (CD4 versus CD8 plot)
7. Analysis of activations markers (SSC-A versus CD40L [CD154], interleukin-2, -13, -17, tumor necrosis factor α, interferon γ and 4-1BB [CD137], respectively) was made on both CD4+CD8- T cells and CD8+CD4- T cells. In this step, positive signals were distinguished from background signals by a gating based on (i) the background response obtained from each sample (medium stimulation condition), and (ii) the positive sample control put on each plate.

**Statistical analyses**

***Sample size calculations***

We aimed to enroll 38 participants each in groups B1 and C1, and 19 each in groups B2, B3, and C2 to obtain 30 evaluable patients in groups B1 and C1, and 15 in the other three groups (assuming 20% non-evaluable participants) for the efficacy proof-of-principle analysis. Assuming that 30% of participants would have HBsAg loss or ≥1-log decrease in qHBsAg, this sample size would give at least 70% power to conclude on a vaccine effect (i.e., lower limit of 80% CI on the percentage of responders ≥15%) at a one-sided alpha level of 10%. In addition, with a standard deviation between 0.5 and 0.9 IU/mL for the HBsAg concentration, it would provide at least 97% power to demonstrate a 1-log difference in mean HBsAg concentration between a vaccine group and its control, at a one-sided alpha level of 10%.

For the ancillary study, we planned to enroll the 76 participants randomized in Step B of the main study. If all quantitative PCR tests performed on immunized patients were negative for ChAd155 vector DNA for ≥3 consecutive days, we would be able to rule out a shedding rate above 9.3% (i.e., upper limit of two-sided 95% CI >9.3%) with 97.5% confidence.

***Changes to the planned analyses***

Because of the early termination of the study (due to lack of efficacy), the following main changes were implemented compared to the planned analyses.

- The final efficacy and immunogenicity analyses originally planned from day 505 up to day 841 were canceled due to the early termination of the study on 8 October 2024. However, samples that had already been tested or processed for testing were still analyzed.
- Primary analyses of efficacy and immunogenicity were performed on the modified per-protocol sets instead of the per-protocol sets. The modified sets extended the maximum interval between subsequent vaccination up to 111 days, which is consistent with study procedures during special circumstances.
- Anti-HBc testing was conducted for a subset of participants only (10 from B2 and 14 from C1). Geometric mean concentrations and seropositivity rates with exact 95% CIs were tabulated.

***Handling of missing data***

For a given participant and a given immunogenicity measurement, missing or non-evaluable measurements were not replaced. Participants who missed reporting solicited/unsolicited AEs or concomitant medications were treated as participants without solicited/unsolicited AEs or concomitant medications.

**Supplementary results**

**Investigation of safety signals**

***Cardiac events***

A thorough review of cardiac events reported in the current study and in another study with the same vaccines was conducted. An Integrated Cardiac Safety Panel reviewed blinded clinical data from the two studies on the occurrence of cardiac events. The available clinical details, including medical history, comorbidities, and concomitant medications, and investigations done (e.g., electrocardiograms at various timepoints, cardiac magnetic resonance imaging, cardiac markers) were reviewed in detail by the panel, for all the participants with reported cardiac events. The myocarditis diagnosis of the case reported in the current study could not be confirmed because a biopsy was lacking and diagnostic criteria of the European Society of Cardiology were not fulfilled. The participant was asymptomatic and exhibited normal cardiac enzymes, no confirmatory magnetic resonance imaging findings, and non-specific electrocardiogram changes indicative of myocarditis. Other reported cardiac events lacked electrocardiogram confirmation and/or occurred in patients with underlying conditions or confounding factors that could have contributed to the cardiac events. A cumulative search of GSK’s safety and clinical database and a review of the medical literature up to 25 May 2023 did not provide adequate evidence for a causal association between cardiac events and the combination of viral vector HBV vaccines and adjuvanted HBV protein vaccine. As no causal association could be established, the validated signal was considered refuted by GSK.

***Hematuria and proteinuria***

Hematuria and proteinuria events in the current study were identified using urine dipstick test results. Such results are prone to error and have limited reliability, especially when read after longer-than-recommended development times, at low concentrations, or when detecting trace amounts. Study participants had a long history of chronic hepatitis B, were on long-term nucleo(s)tide analog therapy, and many had pre-existing conditions (e.g., diabetes, hypertension, dyslipidemia). Up to 45% of participants had proteinuria or hematuria at baseline. Comorbid conditions like diabetes and hypertension are associated with renal diseases, proteinuria, and hematuria. This may have contributed to the high number of abnormalities identified in urinalysis and confounded the interpretation of these abnormalities. The hematuria and proteinuria events occurring during the study were generally low-grade and transient, and resolved without medical intervention. They showed no consistent pattern of sustained or worsening kidney injury based on creatinine or glomerular filtration rate measurements. In participants with pre-existing hematuria and proteinuria, no exacerbation was identified.

**Supplementary references**

1.	Leroux-Roels I, De Coster I, Borobia AM, Langley JM, Ochoa Mazarro D, Ruiz-Antorán B, et al. Safety and immunogenicity of an investigational mRNA-lipid nanoparticle-based monovalent influenza vaccine: Results from a phase 1, randomized, dose-escalation study. Hum Vaccin Immunother. 2026;22:2631804.

**Supplementary tables**

**Table S1. Number and percentage of participants reporting adverse events within 6 months post-immunization (exposed set)**

|  | **Step A** | | | **Step B** | | | **Step C** | |
| --- | --- | --- | --- | --- | --- | --- | --- | --- |
| **Type of AE** | **A1**  **(N=4)** | **A2**  **(N=4)** | **A3**  **(N=5)** | **B1**  **(N=39)** | **B2**  **(N=18)** | **B3**  **(N=19)** | **C1**  **(N=27)** | **C2**  **(N=18)** |
| Medically attended | 2 (**50.0**) | 2 (**50.0**) | 1 (**20.0**) | 14 (**35.9**) | 8 (**44.4**) | 6 (**31.6**) | 11 (**40.7**) | 6 (**33.3**) |
| Any SAE | 0 (**0.0**) | 0 (**0.0**) | 0 (**0.0**) | 1 (**2.6**) | 0 (**0.0**) | 0 (**0.0**) | 1 (**3.7**) | 1 (**5.6**) |
| Any pIMD | 0 (**0.0**) | 0 (**0.0**) | 0 (**0.0**) | 0 (**0.0**) | 0 (**0.0**) | 0 (**0.0**) | 1 (**3.7**) | 0 (**0.0**) |
| Liver disease AESI | 0 (**0.0**) | 0 (**0.0**) | 0 (**0.0**) | 0 (**0.0**) | 0 (**0.0**) | 0 (**0.0**) | 2 (**7.4**) | 0 (**0.0**) |
| Hematological AESI | 0 (**0.0**) | 0 (**0.0**) | 0 (**0.0**) | 0 (**0.0**) | 0 (**0.0**) | 0 (**0.0**) | 1 (**3.7**) | 0 (**0.0**) |

Interventions administered: A1, low doses of ChAd155-hIi-HBV (prime) on day 1, MVA-HBV (boost) on day 57, and HBc-HBs/AS01_B_ on days 113 and 169; A2, four low doses of HBc-HBs/AS01_B_ (days 1, 57, 113, and 169); A3, four placebo doses (days 1, 57, 113, and 169); B1, high doses of ChAd155-hIi-HBV (prime) on day 1, MVA-HBV (boost) on day 57, and HBc-HBs/AS01B on days 113 and 169; B2, four high doses of HBc-HBs/AS01_B_ (days 1, 57, 113, and 169); B3, two placebo doses (days 1 and 57), followed by high doses of ChAd155-hIi-HBV (prime) on day 113 and MVA-HBV (boost) on day 169; C1, high dose of ChAd155-hIi-HBV (prime) on day 1 followed by three high doses of MVA-HBV (boost) on days 57, 113, and 169, with all four doses co-administered with high doses of HBc-HBs/AS01_B_; C2, two placebo doses (days 1 and 57), followed by high doses of ChAd155-hIi-HBV (prime) on day 113 and MVA-HBV (boost) on day 169, both co-administered with high doses of HBc-HBs/AS01_B_. AE, adverse event; AESI, adverse event of special interest; N, number of participants in the exposed set; pIMD, potential immune-mediated disease; SAE, serious adverse event.

**Table S2. Percentage of participants with at least 1-log decrease in qHBsAg or HBsAg loss at day 337 compared to day 1 (modified per-protocol set for efficacy)**

|  | **qHBsAg ≥1-log decrease or HBsAg loss, % (80% CI^a^)** | | | | | | | |
| --- | --- | --- | --- | --- | --- | --- | --- | --- |
|  | **Step A**  **(sequential administration low doses)** | | | **Step B**  **(sequential administration high doses)** | | | **Step C**  **(co-administration high doses)** | |
| **Timepoint** | **A1**  **N=2** | **A2**  **N=2** | **A3**  **N=3** | **B1**  **N=32** | **B2**  **N=16** | **B3**  **N=18** | **C1**  **N=20** | **C2**  **N=14** |
| **Day 337** | 0.0 (0.0–68.4) | 0.0 (0.0–68.4) | 0.0 (0.0–53.6) | 0.0 (0.0–6.9) | 0.0 (0.0–13.4) | 0.0 (0.0–12.0) | 0.0 (0.0–10.9) | 0.0 (0.0–15.2) |

Interventions administered: A1, low doses of ChAd155-hIi-HBV (prime) on day 1, MVA-HBV (boost) on day 57, and HBc-HBs/AS01_B_ on days 113 and 169; A2, four low doses of HBc-HBs/AS01_B_ (days 1, 57, 113, and 169); A3, four placebo doses (days 1, 57, 113, and 169); B1, high doses of ChAd155-hIi-HBV (prime) on day 1, MVA-HBV (boost) on day 57, and HBc-HBs/AS01_B_ on days 113 and 169; B2, four high doses of HBc-HBs/AS01_B_ (days 1, 57, 113, and 169); B3, two placebo doses (days 1 and 57), followed by high doses of ChAd155-hIi-HBV (prime) on day 113 and MVA-HBV (boost) on day 169; C1, a high dose of ChAd155-hIi-HBV (prime) on day 1 followed by three high doses of MVA-HBV (boost) on days 57, 113, and 169, with all four doses co-administered with high doses of HBc-HBs/AS01_B_; C2, two placebo doses (days 1 and 57), followed by high doses of ChAd155-hIi-HBV (prime) on day 113 and MVA-HBV (boost) on day 169, both co-administered with high doses of HBc-HBs/AS01_B_. N, number of participants at day 337; qHBsAg, quantitative HBsAg.

^a^Proof-of-principle was achieved if the lower limit of the 80% CI was ≥15%.

**Table S3. Geometric mean of qHBsAg at each visit in Steps B and C^a^ (modified per-protocol set for efficacy)**

|  | **Step B (sequential administration high doses)** | | | | | | **Step C (co-administration high doses)** | | | |
| --- | --- | --- | --- | --- | --- | --- | --- | --- | --- | --- |
| **Timepoint** | **B1** | | **B2** | | **B3** | | **C1** | | **C2** | |
|  | **N** | **GM (95% CI), IU/mL** | **N** | **GM (95% CI), IU/mL** | **N** | **GM (95% CI), IU/mL** | **N** | **GM (95% CI), IU/mL** | **N** | **GM (95% CI), IU/mL** |
| Day 1 | 35 | 1312.8 (708.9–2431.1) | 17 | 794.9 (431.6–1464.0) | 18 | 792.0 (380.8–1647.0) | 23 | 1520.4 (708.3–3264.0) | 18 | 1375.1 (632.8–2988.0) |
| Day 31 | 24 | 1566.8 (754.1–3255.4) | 13 | 945.3 (459.2–1945.9) | 16 | 835.1 (376.1–1854.1) | 10 | 2577.5 (723.0–9188.9) | 7 | 1344.3 (328.0–5510.2) |
| Day 57 | 35 | 1276.3 (682.9–2385.3) | 17 | 789.2 (423.8–1469.9) | 18 | 773.2 (372.5–1604.8) | 22 | 1322.0 (606.5–2881.4) | 15 | 1195.5 (494.7–2889.1) |
| Day 87 | 18 | 1785.5 (855.0–3728.5) | 11 | 1108.0 (509.1–2411.4) | 14 | 958.3 (410.4–2237.5) | 10 | 1574.3 (439.0–5646.3) | 6 | 1115.3 (194.6–6391.0) |
| Day 113 | 33 | 1321.9 (680.1–2569.4) | 16 | 711.7 (369.2–1371.8) | 18 | 746.9 (363.4–1535.2) | 20 | 959.2 (456.8–2014.0) | 15 | 958.2 (423.2–2169.7) |
| Day 143 | 19 | 1947.4 (842.3–4502.2) | 9 | 1104.7 (450.9–2706.5) | 15 | 824.6 (338.4–2009.0) | 7 | 1060.2 (255.4–4400.8) | 5 | 658.0 (118.0–3669.7) |
| Day 169 | 32 | 1207.2 (613.0–2377.3) | 16 | 675.2 (355.1–1283.9) | 18 | 745.3 (355.6–1562.1) | 20 | 951.4 (458.5–1974.0) | 13 | 801.7 (332.2–1934.9) |
| Day 199 | 17 | 2073.1 (878.0–4895.1) | 9 | 1085.1 (428.3–2749.1) | 15 | 808.7 (333.9–1958.3) | 8 | 1208.5 (293.9–4968.7) | 7 | 927.1 (285.1–3014.8) |
| Day 225 | 31 | 1181.8 (594.4–2349.6) | 15 | 570.6 (318.7–1021.9) | 18 | 739.0 (351.3–1554.7) | 20 | 862.1 (404.2–1838.6) | 13 | 735.1 (309.7–1745.1) |
| Day 281 | 30 | 1091.3 (563.1–2115.0) | 15 | 637.5 (315.5–1288.1) | 18 | 737.4 (350.0–1553.7) | 20 | 880.7 (422.1–1837.9) | 14 | 789.1 (357.2–1743.4) |
| Day 337 | 32 | 1207.7 (615.5–2369.7) | 16 | 654.5 (344.9–1242.2) | 18 | 703.1 (331.5–1491.3) | 20 | 906.2 (433.9–1892.8) | 14 | 739.8 (320.9–1705.6) |
| Day 421 | 31 | 1378.8 (707.3–2687.7) | 16 | 708.3 (380.1–1320.0) | 18 | 804.1 (391.3–1652.4) | 20 | 1069.8 (516.2–2217.2) | 14 | 939.1 (417.4–2113.0) |
| Day 505 | 32 | 1132.0 (572.1–2239.9) | 16 | 665.2 (370.9–1193.0) | 18 | 785.0 (382.5–1611.1) | 18 | 1021.1 (492.4–2117.7) | 12 | 951.2 (431.8–2095.2) |

Interventions administered: B1, high doses of ChAd155-hIi-HBV (prime) on day 1, MVA-HBV (boost) on day 57, and HBc-HBs/AS01_B_ on days 113 and 169; B2, four high doses of HBc-HBs/AS01_B_ (days 1, 57, 113, and 169); B3, two placebo doses (days 1 and 57), followed by high doses of ChAd155-hIi-HBV (prime) on day 113 and MVA-HBV (boost) on day 169; C1, a high dose of ChAd155-hIi-HBV (prime) on day 1 followed by three high doses of MVA-HBV (boost) on days 57, 113, and 169, with all four doses co-administered with high doses of HBc-HBs/AS01_B_; C2, two placebo doses (days 1 and 57), followed by high doses of ChAd155-hIi-HBV (prime) on day 113 and MVA-HBV (boost) on day 169, both co-administered with high doses of HBc-HBs/AS01_B_. GM, geometric mean; N, number of participants with available results; qHBsAg, quantitative HBsAg.

^a^Results for the groups in Step A are not shown here because group sizes are too small to provide meaningful results.

**Supplementary figures**

**Figure S1. qHBsAg adjusted geometric mean concentration ratios between the different vaccine and relevant control groups (modified per-protocol set for efficacy)**

GMC ratios with 80% CIs (depicted here as error bars) were computed with an ANCOVA model on the log_10_-transformation of the concentrations; the model included vaccine group and country as fixed effects and pre-vaccination HBsAg concentration as covariate.

Interventions administered: A1, low doses of ChAd155-hIi-HBV (prime) on day 1, MVA-HBV (boost) on day 57, and HBc-HBs/AS01_B_ on days 113 and 169; A2, four low doses of HBc-HBs/AS01_B_ (days 1, 57, 113, and 169); A3, four placebo doses (days 1, 57, 113, and 169); B1, high doses of ChAd155-hIi-HBV (prime) on day 1, MVA-HBV (boost) on day 57, and HBc-HBs/AS01_B_ on days 113 and 169; B2, four high doses of HBc-HBs/AS01_B_ (days 1, 57, 113, and 169); B3, two placebo doses (days 1 and 57), followed by high doses of ChAd155-hIi-HBV (prime) on day 113 and MVA-HBV (boost) on day 169; C1, high dose of ChAd155-hIi-HBV (prime) on day 1 followed by three high doses of MVA-HBV (boost) on days 57, 113, and 169, with all four doses co-administered with high doses of HBc-HBs/AS01_B_; C2, two placebo doses (days 1 and 57), followed by high doses of ChAd155-hIi-HBV (prime) on day 113 and MVA-HBV (boost) on day 169, both co-administered with high doses of HBc-HBs/AS01_B_. D, day; GMC, geometric mean concentration; qHBsAg, quantitative HBsAg.
